# Targeting the cGAS‐STING Pathway Inhibits Peripheral T‐cell Lymphoma Progression and Enhances the Chemotherapeutic Efficacy

**DOI:** 10.1002/advs.202306092

**Published:** 2023-12-25

**Authors:** Xueying Lu, Shunan Wang, Xin Hua, Xiao Chen, Mengtao Zhan, Qiaoyun Hu, Lei Cao, Zijuan Wu, Wei Zhang, Xiaoling Zuo, Renfu Gui, Lei Fan, Jianyong Li, Wenyu Shi, Hui Jin

**Affiliations:** ^1^ Lymphoma Center, Department of Hematology Jiangsu Province Hospital The First Affiliated Hospital of Nanjing Medical University Nanjing 210029 China; ^2^ Key Laboratory of Hematology of Nanjing Medical University Nanjing 210029 China; ^3^ Jiangsu Key Lab of Cancer Biomarkers Prevention, and Treatment Collaborative Innovation Center for Personalized Cancer Medicine Nanjing Medical University Nanjing 210029 China; ^4^ Department of Oncology Affiliated Hospital of Nantong University Nantong 226001 China; ^5^ Nanjing Aoyin Biotechnology Company Limited Nanjing 210043 China; ^6^ Singleron Biotechnologies Nanjing 211899 China; ^7^ Nanjing Pukou Central Hospital PuKou Branch Hospital of Jiangsu Province Hospital Nanjing 211800 China; ^8^ National Clinical Research Center for Hematologic Diseases The First Affiliated Hospital of Soochow University Suzhou 215006 China

**Keywords:** cGAS‐STING pathways, CLK1, DNA damage repairs, peripheral T‐cell lymphoma, single‐cell RNA sequencing

## Abstract

Peripheral T‐cell lymphoma (PTCL) is a highly heterogeneous group of mature T‐cell malignancies. The efficacy of current first‐line treatment is dismal, and novel agents are urgently needed to improve patient outcomes. A close association between the cyclic GMP‐AMP synthase‐stimulator of interferon genes (cGAS‐STING) pathway and tumor promotion exists, revealing prospective therapeutic targets. This study, investigates the role of the cGAS‐STING pathway and its underlying mechanisms in PTCL progression. Single‐cell RNA sequencing showes that the cGAS‐STING pathway is highly expressed and closely associated with PTCL proliferation. cGAS inhibition suppresses tumor growth and impaires DNA damage repair. Moreover, Cdc2‐like kinase 1 (CLK1) is critical for residual tumor cell survival after treatment with cGAS inhibitors, and CLK1 suppression enhances sensitivity to cGAS inhibitors. Single‐cell dynamic transcriptomic analysis indicates reduced proliferation‐associated nascent RNAs as the underlying mechanism. In first‐line therapy, chemotherapy‐triggered DNA damage activates the cGAS‐STING pathway, and cGAS inhibitors can synergize with chemotherapeutic agents to kill tumors. The cGAS‐STING pathway is oncogenic in PTCL, whereas targeting cGAS suppresses tumor growth, and CLK1 may be a sensitivity indicator for cGAS inhibitors. These findings provide a theoretical foundation for optimizing therapeutic strategies for PTCL, especially in patients with relapsed/refractory disease.

## Graphical abstract

The cGAS‐STING pathway is highly expressed and promotes proliferation in PTCL. cGAS inhibition suppresses tumor growth and impairs DNA repair. Cdc2‐like kinase 1 (CLK1) promotes residual tumor cell survival, and CLK1 suppression enhances the sensitivity to cGAS inhibitors by decreasing proliferation‐associated nascent RNAs. Chemotherapy‐triggered DNA damage activated the cGAS‐STING pathway and cGAS inhibitors synergize with chemotherapeutics in tumor killing.

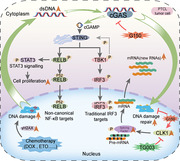

## 1 Introduction

Peripheral T‐cell lymphoma (PTCL) is a group of highly heterogeneous lymphoid malignancies originating from mature T lymphocytes, accounting for 10–15% of non‐Hodgkin's lymphomas. PTCL encompasses nearly 30 distinct subtypes, including angioimmunoblastic T‐cell lymphoma (AITL) and PTCL‐not otherwise specified (PTCL‐NOS), the two most frequent subtypes accounting for over 50% of PTCL cases.^[^ ^1^ ^]^ The clinical manifestations of PTCL are often dominated by an inflammatory phenotype, including skin rash, allergy, fever, and autoimmune hemolytic anemia; a higher level of inflammatory markers implies a worse prognosis.^[^ ^2^, ^3^, ^4^ ^]^ The molecular mechanism underlying the high invasiveness and rapid progression of PTCL remains unidentified due to its complex clinicopathological characteristics and diagnostic difficulties.^[^ ^5^, ^6^ ^]^ Moreover, traditional chemotherapy remains the first‐line treatment for PTCL; however, recurrence and progression rates are extremely high. Therefore, new drugs and therapeutic strategies are needed to improve the prognosis of patients with PTCL, particularly those with relapsed/refractory (RR) disease.

The cyclic GMP‐AMP synthase‐stimulator of interferon genes (cGAS‐STING) signaling pathway is a cytosolic double‐stranded DNA (dsDNA)‐sensing pathway associated with inflammation and autoimmunity. cGAS‐STING triggers the innate immune response and interferon signaling, inducing inflammatory cytokines and interferon production to defend against microbial pathogen invasion. The cGAS‐STING pathway plays a vital role in antitumor immunity and, thus, may be a promising pharmacological target for immunotherapy.^[^ ^7^, ^8^, ^9^ ^]^ Several STING agonists have been investigated in clinical trials; however, their results have been unsatisfactory.^[^ ^10^, ^11^, ^12^ ^]^ Mounting evidence suggests that the cGAS‐STING pathway promotes tumor metastasis and induces an immunosuppressive tumor microenvironment (TME).^[^ ^13^, ^14^, ^15^ ^]^ Although the cGAS‐STING pathway has a dual function in tumors, its role in PTCL remains unclear.

In this study, we aimed to investigate the underlying molecular mechanism, efficiency, potential sensitivity indicators, and a prospective combination regimen of cGAS inhibitors. We demonstrated for the first time that the activated cGAS‐STING pathway plays an oncogenic role in PTCL progression.

## 2 Results

### 2.1 Single‐cell RNA Sequencing Reveals a High cGAS/STING Expression in RR AITL and its Direct Correlation with Proliferation

AITL is a major PTCL subtype characterized by intense inflammation and immune reactions, and an elevated C‐reactive protein level is a poor independent prognostic factor for AITL.^[^ ^16^, ^17^ ^]^ Single‐cell RNA sequencing (scRNA‐seq) is widely used to study hematological malignancies because of its extraordinary advantages in exploring cellular heterogeneity.^[^ ^18^, ^19^, ^20^, ^21^ ^]^ We performed scRNA‐seq to examine the cellular identity and investigate the expression heterogeneity of the cGAS‐STING pathway, which is closely associated with inflammation and immunity in AITL.

**Figure 1:**
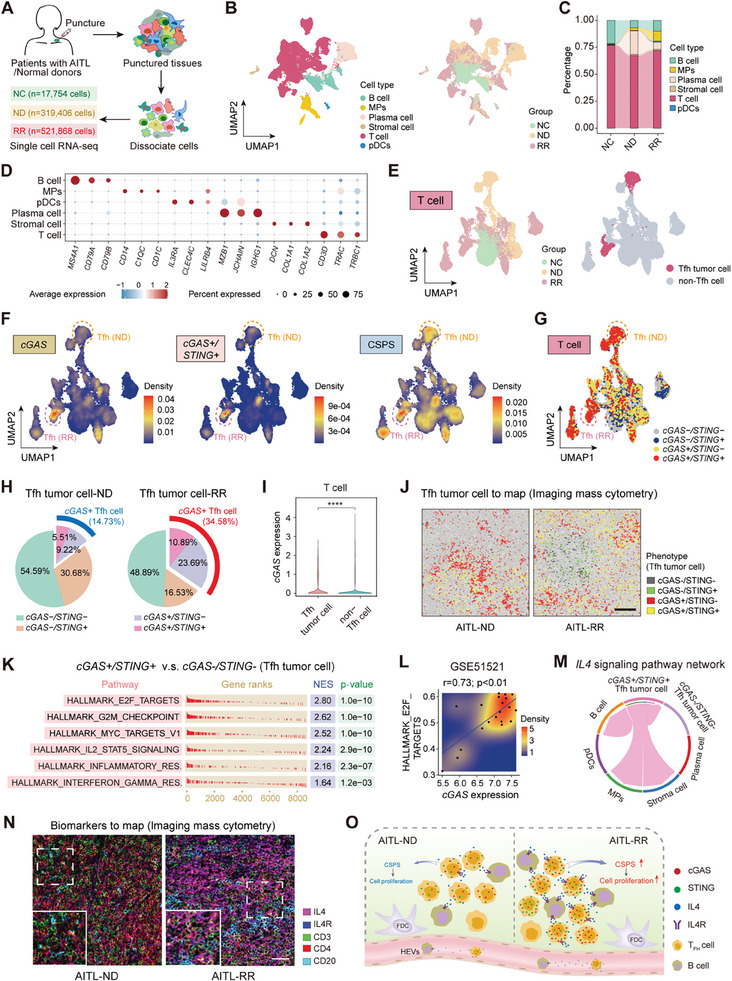
Expression and function of the cGAS‐STING pathway in AITL as detected by single‐cell RNA sequencing. A) Schematic overview of the experimental design. Single‐cell RNA sequencing of ND‐, RR‐AITL, and NC specimens. B) UMAP plots of 859028 cells showing six cell types (left) and three groups (NC, ND, and RR) (right) in nine specimens. C) Alluvial plot showing the proportion of different cell types in the three groups. D) Dot plot showing the expression of three representative marker genes in each cell type. E) UMAP plots of 34773 T cells showing three groups (NC, ND, and RR) (left) and the distribution of Tfh tumor cells (*n =* 4088, *n =* 2509 in cluster 3 in the top right, *n =* 1579 in cluster 11 and 23 on the bottom left) and non‐Tfh cells (right). F) The density of *cGAS+* cells, *cGAS+/STING+* cells, and CSPS shown in the UMAP visualization of T and Tfh tumor cells are marked in dashed circles. G) Four phenotypes (*cGAS+/STING+, cGAS+/STING‐, cGAS‐/STING+*, and *cGAS‐/STING‐*) shown in the UMAP visualization of T cells, and Tfh tumor cells are marked in dashed circles. H) Pie charts showing the proportion of four phenotypes (*cGAS+/STING+* [*n =* 862]*, cGAS+/STING‐* [*n =* 2534]*, cGAS‐/STING+* [*n =* 3384], and *cGAS‐/STING‐* [*n =* 22027]) in Tfh tumor cells of the ND (*n =* 2506) and RR (*n =* 1579) groups. I) Violin plot showing the *cGAS* expression in Tfh tumor cells (*n =* 4088, average cGAS expressio*n =* 0.26) and non‐Tfh cells (*n =* 30685, average cGAS expressio*n =* 0.15). J) Images of imaging mass cytometry showing the distribution of four phenotypes (*cGAS+/STING+, cGAS+/STING‐, cGAS‐/STING+*, and *cGAS‐/STING*‐) in Tfh tumor cells of ND‐ and RR‐AITL specimens. Tfh tumor cells were identified by co‐expressing CD4 and at least two markers in CD10, PD1, and BCL6. Scale bar, 200 µm. K) Hallmark analysis of increasingly expressed genes showing enriched pathways in *cGAS+/STING+* Tfh tumor cells compared with *cGAS‐/STING‐* Tfh tumor cells. L) Scatterplot with density showing the correlation between *cGAS* expression and E2F targets pathway in bulk RNA sequencing data of PTCL (GSE160119). M) Chord diagram showing the interaction between tumor and other cells via IL4/IL4R pair with Cellchat. N) Images of imaging mass cytometry showing IL4, IL4R, CD3, CD4, and CD20 expression in ND‐ and RR‐AITL specimens. Scale bar, 200 µm. O) Summary of the oncogenic role of the cGAS‐STING pathway and the interaction between Tfh tumor cells and B cells via IL4/IL4R repair. **** *p<*0.0001. The Wilcoxon test was used to detect differences between groups (R language). AITL, angioimmunoblastic T cell lymphoma; UMAP, Uniform Manifold Approximation and Projection; cGAS, cyclic GMP‐AMP synthase; STING, stimulator of interferon genes; cGAS‐STING, cyclic GMP‐AMP synthase‐stimulator of interferon genes; CSPS, cGAS‐STING pathway score; NC, normal lymph node; ND, newly diagnosed; RR, relapsed/refractory; IL4, interleukin‐4; IL4R, IL4 receptor; MPs, mononuclear phagocytes; pDCs, plasmacytoid dendritic cells; FDC, follicular dendritic cells; HEVs, high endothelial venules.

Lymph node biopsies from one, three, and five patients with normal lymph nodes (NC group), newly diagnosed AITL (ND group), and RR AITL (RR group), respectively, were collected for scRNA‐seq (**Figure** **1**A). The baseline characteristics of the samples used for scRNA‐seq are shown in Table S1 (Supporting Information). In total, 859 028 cells were captured, including six cell types: T cells, B cells, mononuclear phagocytes, plasma cells, stromal cells, and plasmacytoid dendritic cells (Figure 1B–D). T cells were the major components of the TME (Figure S1A, Supporting Information). We further classified T cells into 24 clusters, and the main clusters varied among the NC, ND, and RR groups (Figure S1B,C, Supporting Information). T follicular helper (Tfh) cells, the cell origin of AITL, were identified by the expression of seven marker genes, and the Tfh score was constructed accordingly (Figure S1D, Supporting Information). The T cell clusters with the top three Tfh scores (clusters 11, 23, and 3) were identified as Tfh tumor cells (Figure 1E and Figure S1E, Supporting Information). Subsequently, T cells were divided into four groups according to cGAS and STING expression: cGAS‐positive/STING‐negative (*cGAS+/STING*‐), cGAS‐negative/STING‐positive (*cGAS‐/STING*+), cGAS‐positive/STING‐positive (*cGAS+/STING*+), and cGAS‐negative/STING‐negative (*cGAS‐/STING*‐). We then calculated the cGAS‐STING pathway score (CSPS) based on the expression of the seven marker genes in this pathway (Figure S1F, Supporting Information). The enrichment levels of *cGAS, cGAS+/STING+*, and CSPS were higher in Tfh tumor cells than in non‐Tfh cells and the RR group than in the ND group (Figure 1F–J and Figure S1H–K, Supporting Information). cGAS expression levels and CSPS were the highest in T cells of the RR group and were higher in the ND group than in the NC group (Figure S1H,I, Supporting Information). cGAS expression levels and CSPS were positively associated with the Tfh score, as validated in the scRNA‐seq and Gene Expression Omnibus (GEO) datasets (Figure S1L,M, Supporting Information). cGAS expression was positively correlated with STING expression (Figure S1N,O, Supporting Information). Gene set enrichment analysis (GSEA) using MSigDB Hallmark gene sets revealed that *cGAS+/STING*+ Tfh tumor cells were predominantly enriched in cell proliferation‐ and inflammation‐associated pathways compared to *cGAS/STING‐* Tfh tumor cells.^[^ ^22^ ^]^ The E2F target, G2M checkpoint, and MYC target pathways were associated with cell proliferation, whereas interleukin 2 (IL2)‐STAT5 signaling and inflammatory and interferon γ (IFN‐ γ) response pathways were associated with inflammatory response, implying that cGAS‐STING pathway activation might be responsible for the inflammation‐associated symptoms in patients with AITL (Figure 1K). Analyses of the GEO51521 and GEO160119 datasets confirmed the positive correlation between cGAS and these enriched pathways (Figure 1L, Figure S1P–R, Supporting Information). CSPS also correlated with proliferation‐associated pathways (Figure S1S, Supporting Information). cGAS and CSPS expression levels were markedly associated with T cell exhaustion scores (Figure S1T, Supporting Information). The T cell exhaustion score was the highest in the T cells of the *cGAS+/STING*+ group and was higher in the *cGAS+/STING*‐ and *cGAS‐/STING+* groups than in the *cGAS‐/STING*‐ group (Figure S1U, Supporting Information).

We performed intercellular interaction analyses using CellChat to dissect cell‐cell communication. The interaction between tumor cells and other cell types via the interleukin‐4 (IL4)/IL4 receptor (IL4R) pair was considerably enriched in *cGAS+/STING*+ Tfh tumor cells (Figure 1M). IL4 was expressed in T cells, whereas IL4R was expressed in T and B cells. IL4 and IL4R expression was higher in Tfh tumor cells in the RR group, whereas IL‐4+/CD4+ cells and IL‐4R+/CD20+ cells showed cell communication (Figure 1N, Figure S1V–Y, Supporting Information). Figure 1O summarizes the oncogenic role of the cGAS‐STING pathway and the interactions between Tfh tumor and B cells in AITL.

### 2.2 cGAS/STING is Overexpressed in PTCL with High Heterogeneity and Could Be Used as a Prognostic Marker

**Figure 2:**
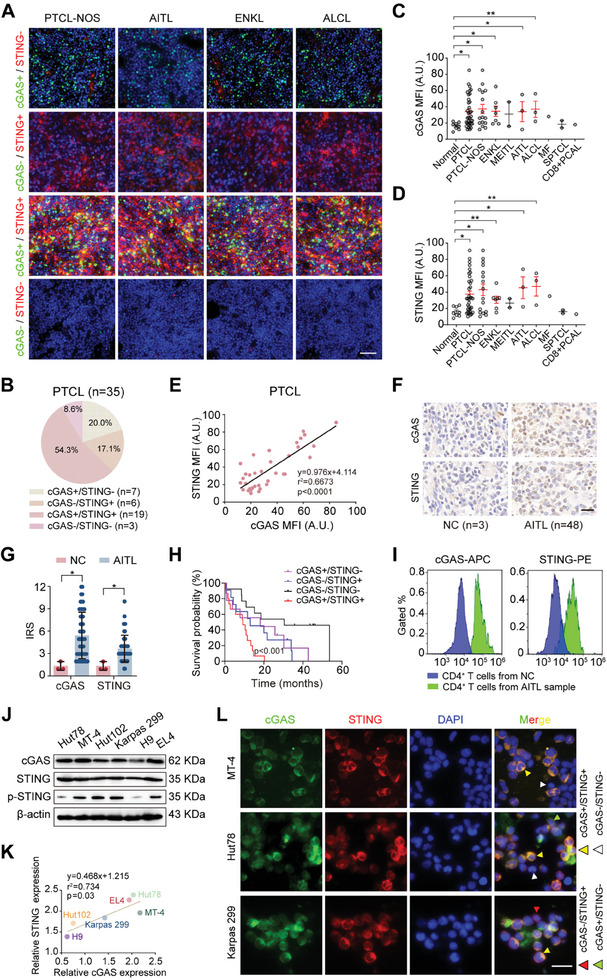
cGAS/STING is overexpressed in PTCL with high heterogeneity and could be used as a prognostic marker. A) cGAS/STING expression in different PTCL subtypes detected by immunofluorescence combined with tissue microarray. Scale bar, 200 µm; green, cGAS; red, STING; blue, nucleus. B) Pie chart showing the percentage of four phenotypes (*cGAS+/STING+, cGAS+/STING‐, cGAS‐/STING+*, and *cGAS‐/STING*‐) in all PTCL samples. C, D) MFI of cGAS (C) or STING (D) in PTCL samples and NCs. Normal (*n =* 8), PTCL (*n =* 35), PTCL‐NOS (*n =* 16), ENKT (*n =* 7), MEITL (*n =* 2), AITL (*n =* 3), ALCL (*n =* 3), MF (*n =* 1), SPTCL (*n =* 2), CD8+ PCAL (*n =* 1). E) Scatterplot showing the correlation between cGAS and STING expression in PTCL samples with mean fluorescence intensity (*n =* 35). F) Representative images of immunohistochemical staining detecting cGAS and STING expression in AITL samples and NCs. Scale bar, 100 µm. NC (*n =* 3), AITL (*n =* 48). G) Quantification analysis of cGAS and STING expression via IRS in AITL samples and NCs. H) Survival analysis showing the overall survival of 48 patients with AITL with four phenotypes (*cGAS+/STING+, cGAS+/STING‐, cGAS‐/STING+*, and *cGAS‐/STING*‐). I) Representative figure of cGAS/STING expression in CD4+ T cells sorted from one normal lymph node (NC) and one AITL sample using flow cytometry (NC [*n =* 1], AITL [*n =* 1]). J) cGAS and STING expression in five human PTCL cell lines (Hut78, MT‐4, Hut102, Karpas 299, H9, and EL4) and one mouse TCL cell line (EL4). K) Scatterplot showing the correlation between cGAS and STING expression in six cell lines (Hut78, MT‐4, Hut102, Karpas 299, H9, and EL4). L) Immunofluorescence showing the heterogeneity of cGAS and STING expression in three PTCL cell lines (MT‐4, Hut78, and Karpas 299). Scale bar, 50 µm; green, cGAS; red, STING; blue, DAPI; yellow triangle, *cGAS+/STING*+; green triangle, *cGAS+/STING*‐; red triangle, *cGAS‐/STING*+; white triangle, *cGAS‐/STING*‐. Experiments were conducted in triplicate. *p<0.05, **p<0.01. Student's t‐test was used to detect differences between groups (GraphPad Prism 9.3). cGAS, cyclic GMP‐AMP synthase; STING, stimulator of interferon genes; NC, normal lymph node; PTCL, peripheral T‐cell lymphoma; PTCL‐NOS, PTCL‐not otherwise specified; AITL, angioimmunoblastic T cell lymphoma; ENKL, extranodal NK/T‐cell lymphoma; ALCL, anaplastic lymphoma kinase positive anaplastic large cell lymphoma; MEITL, monomorphic epitheliotropic T‐cell lymphoma; MF, mycosis fungoides; SPTCL, subcutaneous panniculitic T‐cell lymphoma; PCAL, primary cutaneous acral lymphoma; MFI, mean fluorescence intensity; A.U, arbitrary unit; IRS, immunoreactivity score; APC, allophycocyanin; PE, phycoerythrin; p‐STING, phosphorylated STING; DAPI, 2‐(4‐amidinophenyl)−6‐indolecarbamidine dihydrochloride; WB, western blotting.

Immunofluorescence was performed to detect cGAS and STING expression in a PTCL tissue microarray containing 35 PTCL samples and eight normal lymph nodes (**Figure** **2**A). The distribution of PTCL subtypes is summarized in Figure S2A (Supporting Information). cGAS expression was pronounced in PTCL samples (74.3%), with cGAS+/STING+ cells accounting for the highest proportion (54.3%) (Figure 2B). cGAS and STING expression was higher in PTCL than in normal lymph nodes (Figure 2C,D). Furthermore, cGAS expression was positively correlated with STING expression in PTCL (Figure 2E), including PTCL‐NOS and other subtypes (Figure S2B, Supporting Information). Fifty‐one AITL and three normal lymph node sections were detected using immunohistochemical (IHC) staining. The cGAS and STING expression results were consistent with those of the tissue microarray (Figure 2F,G). The overall survival of patients with the cGAS+/STING+ phenotype was lower than that of patients with cGAS+/STING‐, cGAS‐/STING+, or cGAS‐/STING‐ phenotypes, indicating the prognostic value of cGAS/STING expression (Figure 2H). CD4+ T cells were sorted from three AITL samples and three normal lymph nodes for flow cytometric analysis, and cGAS and STING expression levels were higher in AITL (Figure 2I, Figure S2C,D, Supporting Information). Western blotting (WB) showed that the cGAS‐STING pathway was activated in five human PTCL cell lines (Hut78, MT‐4, Hut102, Karpas 299, and H9) and one murine T‐cell lymphoma (TCL) cell line (EL4) (Figure 2J). Correlation analysis revealed a positive correlation between cGAS and STING expression (Figure 2K). Immunofluorescence showed high heterogeneity in cGAS/STING expression in PTCL cell lines (Figure 2L).

### 2.3 Targeting the cGAS‐STING Pathway Inhibits PTCL Proliferation and Induces Apoptosis

**Figure 3:**
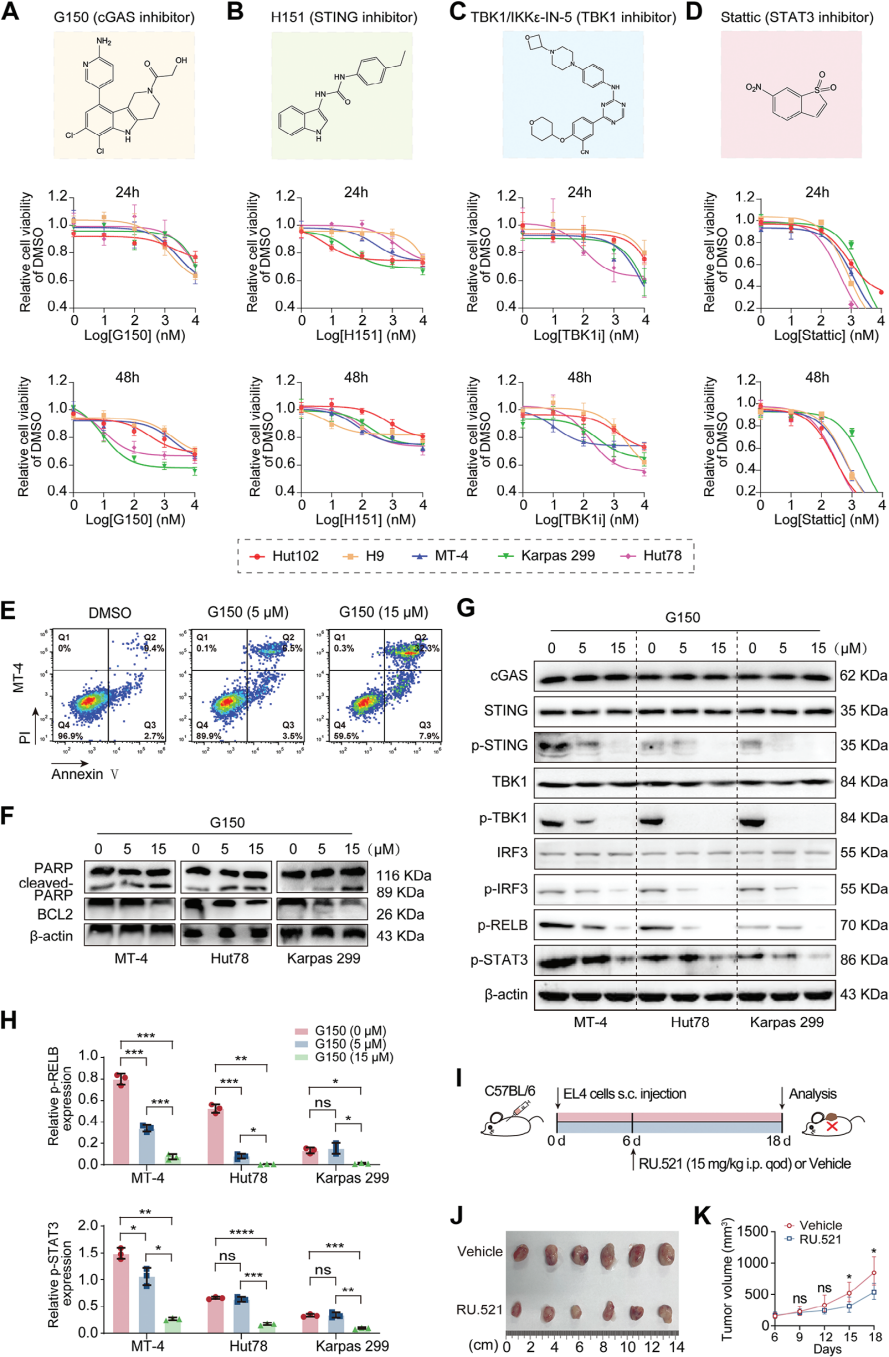
Targeting the cGAS‐STING pathway inhibits PTCL proliferation and induces apoptosis. A–D) The CCK8 assay results show the proliferation levels of PTCL cell lines (Hut102, H9, MT‐4, Karpas 299, and Hut78) after treatment with cGAS‐STING pathway inhibitors (cGAS inhibitor G150, STING inhibitor H151, TBK1 inhibitor TBK1/Ikkε‐IN‐5, and STAT3 inhibitor Stattic) for 24 or 48 h. E) The flow cytometry results show the apoptosis levels of the PTCL cell line (MT‐4) treated with G150 or DMSO for 48 h. F) WB results showing the expression of apoptosis‐associated protein in three PTCL cell lines (MT‐4, Hut78, and Karpas 299) treated with G150 for 48 h. G) The WB results show the expression of cGAS‐STING pathway‐associated protein in three PTCL cell lines (MT‐4, Hut78, and Karpas 299) treated with G150 for 48 h. H) Quantification analyses showing p‐RELB and p‐STAT3 expression in (G). I) Experimental design of T cell lymphoma‐bearing C57BL/6 mice treated with RU.521 or vehicle in vivo. J, K) Tumor volume (J) and tumor growth curve (K) of the RU.521 administration and control groups. (*n =* 6). In vitro experiments were conducted in triplicate. *p<0.05, **p<0.01, ***p<0.001, ****p<0.0001, ns: not significant. Student's t‐test was used to detect differences between groups (GraphPad Prism 9.3). cGAS, cyclic GMP‐AMP synthase; STING, stimulator of interferon genes; TBK1, TANK binding kinase‐1; STAT3, signal transducer and activator of transcription 3; DMSO, dimethyl sulfoxide; PI, propidium iodide; BCL2, B‐cell lymphoma‐2; PARP, poly ADP‐ribose polymerase; p‐STING, phosphorylated STING; p‐TBK1, phosphorylated TBK1; p‐IRF3, phosphorylated interferon regulatory factor 3; p‐RELB, phosphorylated RELB; p‐STAT3, phosphorylated STAT3; s.c., subcutaneously; i.p., intraperitoneal injection; qod, every other day; WB, western blotting.

We assessed the tumor‐killing activity of cGAS‐STING pathway inhibitors because high cGAS/STING expression was correlated with poor outcomes in patients with PTCL. We tested five drugs, including cGAS inhibitors G150 and G140, STING inhibitor H151, TANK binding kinase‐1 (TBK1) inhibitor TBK1/Ikkε‐IN‐5, and signal transducer and activator of transcription 3 (STAT3) inhibitor Stattic. Owing to domain conservation across species, G150/G140 and RU.521 primarily inhibited cGAS activity in humans and mice, respectively. We simultaneously tested RU.521 in a murine T‐cell lymphoma cell line (EL4) (Figure S3A, Supporting Information). The CCK8 assay results demonstrated that these inhibitors suppressed cell proliferation in a concentration‐ and time‐dependent manner (**Figure** **3**A–D and S3B, Supporting Information). The ineffectiveness of the STING agonist diABZI further confirmed the tumorigenic role of the cGAS‐STING pathway in PTCL (Figure S3C, Supporting Information). G150 treatment induced PTCL cell apoptosis concentration‐dependently (Figure 3E and Figure S3D, Supporting Information), which was subsequently verified by WB, showing increased expression of apoptosis‐related proteins (cleaved poly ADP‐ribose polymerase and cleaved‐caspase3) and decreased anti‐apoptosis protein BCL2 expression (Figure 3F). Similar results for apoptosis were obtained following cGAS knockdown (Figure S3E, Supporting Information). The expression levels of STING/TBK1/interferon regulatory factor 3 (IRF3) and their corresponding phosphorylated proteins were detected by WB. p‐STING/p‐TBK1/p‐IRF3 expression decreased concentration‐dependently (Figure 3G). Similar effects were observed after G140 administration (Figure S3F, Supporting Information). A previous study reported that the cGAS‐STING pathway activates the canonical/non‐canonical NF‐κB and STAT3 pathways to promote tumor cell proliferation.^[^ ^23^ ^]^ G150‐treated PTCL cells showed decreased p‐STAT3 and p‐RELB expression compared to the negative control (Figure 3G,H; Figure S3F,G, Supporting Information). In addition, suspensions of TCL cell line EL4 were implanted into immunocompetent C57BL/6 mice via subcutaneous injection to evaluate the effect of the cGAS inhibitor RU.521 on tumor suppression and to exclude immune system interference (Figure 3I). RU.521 monotherapy resulted in markedly tumor shrinkage compared to the control, with no apparent weight loss (Figure 3J,K; Figure S3H, Supporting Information). However, the antitumor effect of RU.521 monotherapy was not strong enough to eradicate tumor cells, and further work was demanded to improve the efficacy.

### 2.4 Targeting cGAS Attenuates DNA Damage Repair in Human PTCL Cells

**Figure 4:**
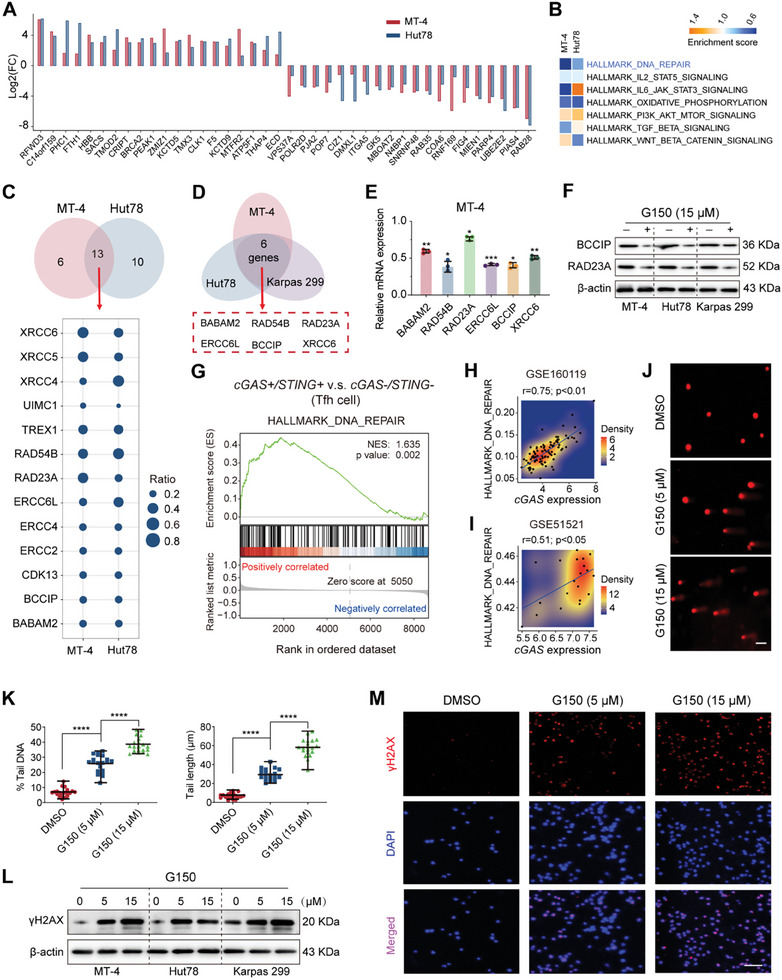
Targeting cGAS attenuates DNA damage repair. A) Data‐independent acquisition analysis of protein mass spectrometry data showing the top 20 up‐ and downregulated proteins in G150‐treated PTCL cell lines (MT‐4 and Hut78). B) Hallmark analyses of differentially expressed protein showing enriched pathways in G150‐treated PTCL cell lines (MT‐4 and Hut78) compared to the control group. C) Venn diagram and dot plot showing 13 downregulated DNA repair‐related proteins in PTCL cell lines (MT‐4 and Hut78). D) Venn diagram showing the six DNA repair‐related genes downregulated in all three G150‐treated PTCL cell lines (MT‐4, Hut78, and Karpas 299) compared with the control group. E) qPCR results show the downregulation of the six DNA repair‐related genes in (D) in the PTCL cell line (MT‐4). F) WB results showing the expression of two representative DNA repair‐related proteins in PTCL cell lines treated with G150 for 48 h. G) Gene set enrichment analysis (GSEA) plot showing the enrichment of DNA repair pathway in *cGAS+/STING+* Tfh tumor cells compared with *cGAS‐/STING‐* Tfh tumor cells with scRNA‐seq data. H, I) Scatterplot with density showing the correlation between *cGAS* expression and DNA repair pathway in bulk RNA sequencing data of PTCL (GSE160119) (H) and AITL (GSE51521) (I). J) Representative images of the PTCL cell line (Hut78) treated with DMSO or G150 in the comet assay. Scale bar, 50 µm. K) Analyses of the percentage tail DNA and a tail length of DMSO‐ or G150‐treated PTCL cell line (Hut78) in the comet assay (*n =* 20). L) WB results showing γH2AX expression in PTCL cell lines (MT‐4, Hut78, and Karpas 299) treated with G150 for 48 h. M) Representative images of G150‐ or DMSO‐treated PTCL cell line (Hut78) with immunofluorescence (*n =* 50). Red, γH2AX; blue, DAPI; purple, merged; Scale bar, 100 µm. Experiments were conducted in triplicate. * *p<*0.05, ** *p<*0.01, *** *p<*0.001, **** *p<*0.0001. Student's t‐test was used to detect differences between groups (GraphPad Prism 9.3). cGAS, cyclic GMP‐AMP synthase; STING, stimulator of interferon genes; ES, enrichment score; NES, normalized ES; Tfh tumor cell, T follicular helper tumor cell; DMSO, dimethyl sulfoxide; WB, western blotting.

cGAS promotes DNA damage repair by activating the STING‐TBK1 pathway.^[^ ^24^ ^]^ Protein mass spectrometry was used to evaluate protein changes in PTCL cell lines (MT‐4 and Hut78) after G150 treatment to explore the applicability of this mechanism to PTCL. The top 20 up‐and‐downregulated proteins are shown in **Figure** **4**A. Functional enrichment analysis of differentially expressed proteins indicated that the DNA repair pathway was affected (Figure 4B). MT‐4 and Hut78 shared 13 DNA repair‐related proteins, all of which were downregulated (Figure 4C). After detecting the expression of the 13 corresponding genes using quantitative real‐time polymerase chain reaction (qRT‐PCR), we further selected six DNA repair‐related genes with decreased expression in all three PTCL cell lines (MT‐4, Hut78, and Karpas 299) after G150 treatment (Figure 4D,E, and Figure S4A,B, Supporting Information). Two DNA repair‐related proteins (BCCIP and RAD23A) were markedly decreased concentration‐dependently (Figure 4F). Compared with *cGAS‐/STING*‐ Tfh tumor cells, the DNA repair pathway was enriched in *cGAS+/STING*+ Tfh tumor cells according to the scRNA‐seq analysis of AITL (Figure 4G). Consistently, cGAS expression was positively correlated with the DNA repair pathway in the GSE160119 and GSE51521 datasets (Figure 4H,I). Similar results were obtained with CSPS (Figure S4C, Supporting Information). Furthermore, the comet assay revealed that G150 treatment led to concentration‐dependent DNA damage, with a longer comet tail length and higher tail DNA percentage than the control (Figure 4J,K). The expression of γH2AX, a marker of DNA double‐strand breaks, demonstrated increased WB and immunofluorescence after G150 or G140 treatment (Figure 4L,M).

### 2.5 Cdc2‐like Kinase 1 may be a Potential Sensitivity Indicator of cGAS Inhibitor G150 and a Promising Target for Combination Therapy

**Figure 5:**
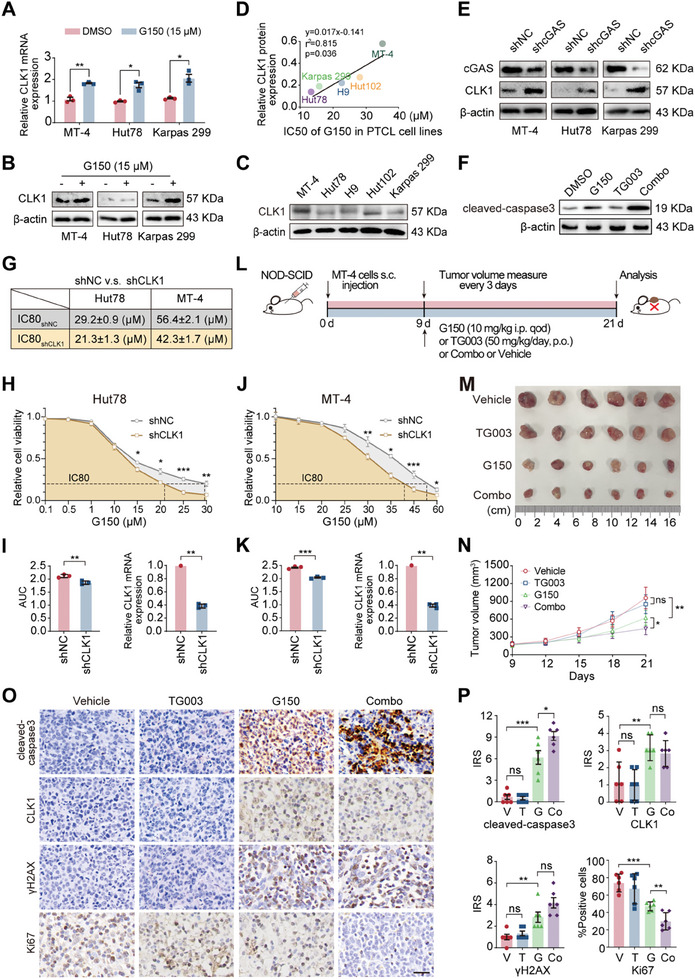
CLK1 may serve as a potential sensitivity indicator of cGAS inhibitor G150 and is a promising target for combination therapy. A) qPCR results showing CLK1 expression in the PTCL cell line (MT‐4, Hut78, and Karpas 299) treated with DMSO or G150 for 48 h. B) WB results showing CLK1 expression in the PTCL cell line (MT‐4, Hut78, and Karpas 299) treated with G150 for 48 h. C) WB results showing CLK1 expression in the PTCL cell line (MT‐4, Hut102, H9, Hut78, and Karpas 299). D) Scatterplot showing the correlation between CLK1 expression and the IC_50_ of G150 in PTCL cell lines (MT‐4, Hut102, H9, Hut78, and Karpas 299). E) WB results showing cGAS and CLK1 expression in PTCL cell lines (MT‐4, Hut78, and Karpas 299) after knocking down cGAS by shRNA (shcGAS) or control (shNC). F) WB results showing the expression of cleaved‐caspase3 in PTCL cell line (MT‐4) treated with G150, TG003, or G150 plus TG003 combination (Combo) therapy for 48 h. G) CCK8 assay results showing the IC_80_ of G150 in PTCL cell lines (Hut78 and MT‐4) after CLK1 was knocked down by shRNA (shCLK1) or control (shNC). H) CCK8 assay results showing the AUC and IC_80_ of G150 in two PTCL cell lines (Hut78) after CLK1 knockdown by shRNA (shCLK1) or control (shNC). I) Quantification of AUC in (H) (left) and qPCR results showing CLK1 expression (right) in a PTCL cell line (Hut78) after CLK1 knockdown by shRNA (shCLK1) or control (shNC). J) CCK8 assay results showing the AUC and IC_80_ of G150 in two PTCL cell lines (MT‐4) after CLK1 knockdown by shRNA (shCLK1) or control (shNC). K) Quantification of AUC in (J) (left) and qPCR results showing CLK1 expression (right) in the PTCL cell line (MT‐4) after CLK1 knockdown by shRNA (shCLK1) or control (shNC). L) Experimental design of PTCL‐bearing NOD‐SCID mice treated with vehicle, TG003, G150, or G150 plus TG003 combination therapy in vivo. M, N) Tumor volume (M) and tumor growth curve (N) of four groups (Vehicle, TG003, G150, and combination therapy). O, P) Representative images of immunohistochemical staining showing cleaved‐caspase3, CLK1, γH2AX, and Ki67 expression in tumor samples of four groups (O) and their IRS (P) (*n =* 6). Scale bar, 100 µm. In vitro experiments were conducted in triplicate. *p<0.05, **p<0.01, ***p<0.001, ns, not significant. Student's t‐test was used to detect differences between groups (GraphPad Prism 9.3). DMSO, dimethyl sulfoxide; CLK1, Cdc2‐like kinase 1; PTCL, peripheral T cell lymphoma; cGAS, cyclic GMP‐AMP synthase; PTCL, peripheral T cell lymphoma; Combo, G150 plus TG003 combination therapy; IC80, 80 percent maximal inhibitory concentration; shCLK1, knock out CLK1 by shRNA; shNC, knocking down nothing by shRNA; AUC, area under curve; IRS, immunoreactivity score; s.c., subcutaneously; i.p., intraperitoneal injection; p.o., by mouth; qod, every other day; V, vehicle; T, TG003; G, G150; Co, G150 plus TG003 combination; WB, western blotting.

Cdc2‐like kinase 1 (CLK1) is a bispecific protein kinase that phosphorylates serine/arginine‐rich splicing factors (SRs), thereby regulating the splicing activity of SRs. This splicing activity controls alternative RNA splicing and influences the ultimate distribution of nascent RNAs in the cytoplasm.^[^ ^25^, ^26^ ^]^ CLK1 was one of the top 20 upregulated proteins in G150‐treated PTCL cells (Figure 4A). Furthermore, qPCR (**Figure** **5**A) and WB (Figure 5B) demonstrated increased CLK1 expression in the G150‐treated PTCL cell lines (MT‐4, Hut78, and Karpas 299). Notably, CLK1 expression was positively associated with the IC_50_ of G150 in PTCL cell lines (Figure 5C,D). cGAS knockdown triggered an increase in CLK1 expression (Figure 5E). Combination treatment with G150 and TG003 induced higher apoptosis‐associated cleaved caspase‐3 expression than treatment with G150 or TG003 alone or the control (Figure 5F). The CCK8 assay indicated a smaller area under the curve and lower IC_80_ values of G150 in shCLK1 PTCL cells than in shNC cells (Figure 5G–K). Thus, CLK1 may be used as a sensitivity indicator of cGAS inhibitors. TG003, a CLK1 inhibitor, had no inhibitory effect on the proliferation of MT‐4 cells (Figure S5A,B, Supporting Information). G150 was synchronized with TG003 to kill PTCL cells (Figure S5C, Supporting Information).

Next, we established a xenograft model to test the antitumor effects of G150 and TG003 as single agents or combination therapy in vivo. MT‐4 cell suspensions were subcutaneously injected into NOD‐SCID mice, which were then treated with G150 and TG003 alone or in combination (Figure 5L). Tumor growth was comparable between control and TG003‐treated mice, indicating the inefficacy of TG003 monotherapy. However, combination therapy resulted in more obvious tumor shrinkage than G150 monotherapy, with no apparent weight loss, implying that TG003 is a G150‐sensitizing agent (Figure 5M,N, and Figure S5D, Supporting Information). Furthermore, Ki67 expression decreased dramatically, whereas cleaved caspase3, γH2AX, and CLK1 expression increased in the combination group with IHC staining (Figure 5O,P).

### 2.6 Single‐Cell Dynamic RNA Sequencing Reveals that TG003 Functions by Inducing Reduced Proliferation‐Related Nascent RNAs based on G150 Treatment

**Figure 6:**
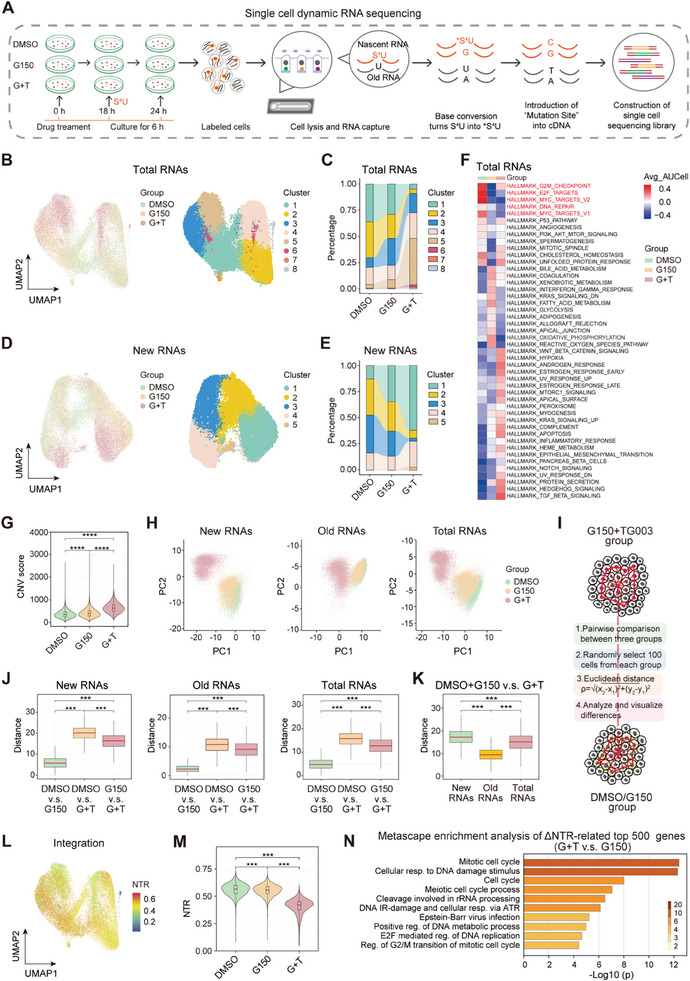
Single‐cell dynamic RNA sequencing reveals that TG003 functions by inducing reduced proliferation‐related nascent RNAs based on G150 treatment. A) Schematic overview of the experimental design. Single‐cell dynamic RNA sequencing of the PTCL cell line (MT‐4) treated with DMSO, G150, or G150 plus TG003 (G+T). B) UMAP plots of 39491 cells showing three groups (left) and eight clusters (right) according to total RNAs (*n =* 3). C) Alluvial plot showing the changes in the proportion of different clusters in three groups according to total RNAs. D) UMAP plots of 39647 cells showing three groups (left) and five clusters (right) according to new RNAs (*n =* 3). E) Alluvial plot showing the changes in the proportion of different clusters in three groups according to new RNAs. F) The heatmap of gene set enrichment analysis of the selected hallmark gene sets in the MSigDB database among the three groups (DMSO, G150, and G150 plus TG003 group) according to total RNAs. G) Violin plot showing the copy number variation score in the three groups (DMSO, G150, and G150 plus TG003 group). H) Principal component (analysis showing the three groups (DMSO, G150, and G150 plus TG003 group) according to new, old, and total RNAs. I) Flow chart calculating the distance among different groups based on (H). J) Box plots showing the pairwise distance among three groups based on new, old, and total RNAs. K) Box plots showing the distance between the DMSO plus G150 and combination groups of G150 and TG003 (G+T) according to new, old, and total RNAs. L) UMAP plot showing the NTR integrating all three groups (DMSO, G150, and G150 plus TG003 group). M) Violin plots showing the NTR of DMSO, G150, and G150 plus TG003 (G+T) group. N) Metascape enrichment analysis of ΔNTR‐related top 500 genes between the G150 plus TG003 (G+T) and G150 groups according to new RNAs. ***p<0.001, ****p<0.0001. The Wilcoxon test was used to detect differences between groups (R language). DMSO, dimethyl sulfoxide; G+T, G150 plus TG003; UMAP, Uniform Manifold Approximation, and Projection; CNV, copy number variation; NTR, new‐to‐total RNA ratio; ΔNTR, NTR difference value.

We used single‐cell dynamic RNA sequencing, a new technology developed by combining dynamic transcriptomes with high‐throughput single‐cell sequencing technology, to investigate how TG003 and G150 synergistically inhibit PTCL cell proliferation.^[^ ^27^, ^28^ ^]^ Moreover, we revealed the dynamic changes in transcripts in PTCL cells by labeling the newly synthesized mRNA to distinguish between old and nascent RNAs after treatment with the three different regimens (**Figure** **6**A). Different groups and cell clusters were obtained according to the total and new RNAs, and their proportions in the combination group were markedly different from those in the other groups, indicating that the transcripts were considerably altered after combination therapy (Figure 6B–E). Trajectory analysis of PTCL cells showed similar results in the control and G150 monotherapy groups, whereas the combination treatment considerably altered the trajectory in the pseudotime analysis based on total and new RNAs (Figure S6A, Supporting Information). The increase in CLK1 expression after being treated with G150 in PTCL cells was further verified using scRNA‐seq (Figure S6B, Supporting Information). Regulatory network analysis was separately performed for new, old, and total RNAs using the Python package transcription factor regulatory network analysis (pySCENIC) and the regulatory specificity score of the selected transcription factors in the three groups (dimethyl sulfoxide (DMSO), G150, and G150 plus TG003 group), identifying the different regulatory statuses of the same regulon in new, old, or total RNAs (Figure S6C, Supporting Information). Proliferation and DNA repair‐related pathways were less enriched in PTCL cells after treatment with G150 according to total RNAs, further confirming our previous results (Figure 6F). The copy number variation score in PTCL cells increased after G150 treatment, and the increase was more pronounced after combination treatment, which may be attributed to further DNA damage (Figure 6G).

Principal component analysis was performed according to new, old, or total RNAs in the three groups, and we designed a method to calculate the distance between the different groups (Figure 6H,I). The distance between the combination group and the other two groups was much greater than that between the DMSO and G150 groups, regardless of new, old, or total RNAs (Figure 6J). The distance between (DMSO + G150) and combination groups based on new RNAs was the longest, whereas the distance according to old RNAs was the shortest, indicating that the distance in total RNAs was primarily attributed to nascent RNAs (Figure 6K). The new‐to‐total RNA ratio (NTR) reflects the degree of new RNA synthesis.^[^ ^29^ ^]^ The NTR of cells in each group was highly heterogeneous (Figure 6L and Figure S6D, Supporting Information). However, the NTR of cells in the combination group was much lower than that in the DMSO and G150 groups, indicating that TG003 inhibits the production of new RNAs (Figure 6M). NTR difference value (ΔNTR, NTR [G150 group] minus NTR [combination group]) was calculated; metascape enrichment analysis of the top 500 largest ΔNTR‐related genes implied an association between nascent RNAs reduction and cell proliferation and DNA repair; thus, adding TG003 enhanced the killing effect of G150 (Figure 6N). The top 500 identified genes are listed in Table S2 (Supporting Information). The expression of the top 100 genes in the new, old, and total RNAs is shown in Figure S6E (Supporting Information).

### 2.7 Chemotherapeutic Agents Can Activate the cGAS‐STING Pathway, and cGAS Inhibition Enhances the Tumor‐Killing Effect in PTCL

**Figure 7:**
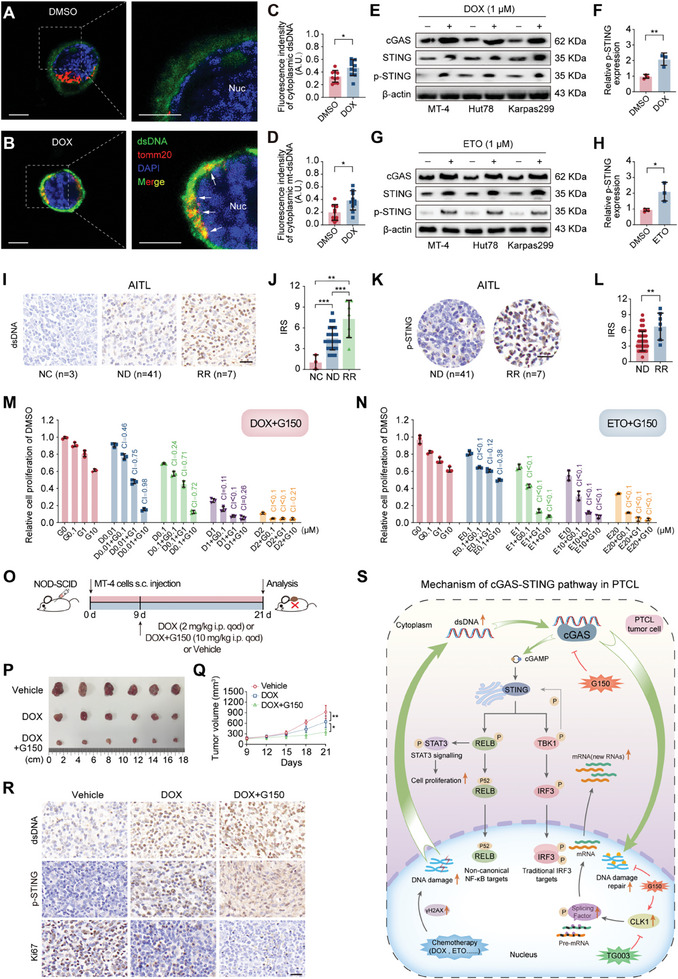
Chemotherapeutic agents could activate the cGAS‐STING pathway, and cGAS inhibition enhances the tumor‐killing effect in PTCL. A, B) Representative confocal images of control (A) and DOX‐treated (B) (1 µM of DOX for 24 h) PTCL cell lines. Green, dsDNA; Red, tomm20; blue, DAPI; scale bar, 10 µm. The dotted square in (A, left) is magnified as (A, right). The dotted square in (B, left) is magnified as (B, right). Arrows in (B, right) highlight mitochondrial dsDNA. C, D) Fluorescence intensity of cytoplasmic (C) and mitochondrial dsDNA (D) in Figure 7A, B (*n =* 10). E) WB results showing cGAS, STING, and p‐STING expression in PTCL cell lines (MT‐4, Hut78, and Karpas 299) treated with 1 µM of doxorubicin for 48 h. F) Quantification analysis showing the p‐STING expression in (E). G) WB results showing cGAS, STING, and p‐STING expression in PTCL cell lines (MT‐4, Hut78, and Karpas 299) treated with 1 µM of ETO for 48 h. H) Quantification analysis showing the p‐STING expression in (G). I) Representative images of immunohistochemical staining showing the dsDNA expression in ND‐, RR‐AITL samples, and NCs. Scale bar, 100 µm. J) Quantification analysis of the dsDNA expression via immunoreactivity score in ND‐, RR‐AITL samples, and NCs. NC (*n =* 3), ND (*n =* 41), RR (*n =* 7). K) Representative images of immunohistochemical staining showing p‐STING expression in ND‐ and RR‐AITL samples. Scale bar, 100 µm. L) Quantification analysis of the p‐STING expression via IRS in ND‐ and RR‐AITL samples. ND (*n =* 41), RR (*n =* 7). M) CCK8 assay results showing the relative proliferation of the PTCL cell line treated with DOX or G150 plus DOX combination treatment for 48 h, with the CI. N) CCK8 assay results show the relative proliferation of the PTCL cell line treated with ETO or G150 plus ETO combination treatment for 48, with the CI. O) Experimental design of human PTCL‐bearing NOD‐SCID mice treated with vehicle, DOX, or DOX plus G150 combination therapy in vivo. P, Q) Tumor volume (P) and tumor growth curve (Q) of three groups (Vehicle, DOX, and DOX plus G150 combination therapy) (*n =* 6). R) Representative images of immunohistochemical staining showing dsDNA, p‐STING, and Ki67 expression in tumor samples of the three groups (Vehicle, DOX, and DOX plus G150 group). Scale bar, 100 µm. S) Mechanism of the cGAS‐STING pathway in PTCL. In vitro experiments were conducted in triplicate. *p<0.05, **p<0.01, ***p<0.001. Student's t‐test was used to detect differences between groups (GraphPad Prism 9.3). Ctrl, control; Nuc, nucleus; dsDNA, double‐stranded DNA; tomm20, mitochondrial 20 kDa outer membrane protein; DAPI, 2‐(4‐amidinophenyl)−6‐indolecarbamidine dihydrochloride; A.U, arbitrary unit; cGAS, cyclic GMP‐AMP synthase; DOX, doxorubicin; ETO, etoposide; STING, stimulator of interferon genes; p‐STING, phosphorylated STING; DMSO, dimethyl sulfoxide; AITL, angioimmunoblastic T‐cell lymphoma; NC, normal lymph node; ND, newly diagnosed; RR, relapsed/refractory; IRS, immunoreactivity score; +, plus; CI, combination index; i.p., intraperitoneal injection; s.c., subcutaneously; qod, every other day; cGAMP, cyclic guanosine monophosphate; TBK1, TANK binding kinase‐1; IRF3, interferon regulatory factor 3; STAT3, signal transducer and activator of transcription 3; PTCL, peripheral T‐cell lymphoma; CLK1, cdc2‐like kinase 1; WB, western blotting.

DNA‐damaging chemotherapy is a key source of cytosolic dsDNA that activates the cGAS‐STING pathway. Doxorubicin (DOX) and etoposide (ETO) are two typical drugs that destroy DNA structure and are the major first‐line treatment options for PTCL. Therefore, the effects of DOX and ETO on the cGAS‐STING pathway were explored in PTCL. Laser confocal microscopy co‐localization revealed that DOX treatment increased cytoplasmic dsDNA, including mitochondrial dsDNA, in PTCL cells compared to control cells (**Figure** **7**A–D). As predicted, the activation of the cGAS‐STING pathway was shown in the WB results (Figure 7E–H). In addition, IHC staining further validated the increase in dsDNA expression and cGAS‐STING pathway activation in RR AITL specimens compared to ND AITL specimens or normal lymph nodes (Figure 7I–L). We calculated the combination index of (G150 plus DOX) and (G150 plus ETO) to investigate whether the cGAS inhibitor could synergize with DOX or ETO to enhance tumor cell killing and observed a synergistic effect in both combination regimens (combination index <1) (Figure 7M,N). Human PTCL‐bearing NOD‐SCID mice were treated with DOX alone or in combination with G150 or DOX (Figure 7O). Adding G150 enhanced tumor shrinkage induced by DOX monotherapy, with no apparent weight loss (Figure 7P,Q, and Figure S7A, Supporting Information). DOX treatment induced dsDNA, p‐STING, and Ki67 upregulation (Figure 7R and Figure S7B, Supporting Information). The mechanism of action of the cGAS‐STING pathway in PTCL is shown in Figure 7S.

## 3 Discussion

The role of the cGAS‐STING pathway in tumors remains controversial. Numerous studies have reported on this pathway, but they primarily focused on solid tumors and the antitumor effects of the pathway.^[^ ^30^, ^31^, ^32^ ^]^ The pro‐tumor effect of the cGAS‐STING pathway has recently been proposed and requires further exploration. However, the role of the cGAS‐STING pathway in PTCL has rarely been reported. The present study is the first to demonstrate that the cGAS‐STING pathway induces PTCL progression and that first‐line chemotherapeutic agents can activate this pathway, potentially leading to relapse or disease progression.

PTCL is characterized by an inflammatory immune microenvironment.^[^ ^33^, ^34^ ^]^ Patients with PTCL often develop inflammatory symptoms and autoimmune diseases, making inflammatory markers good prognostic predictors.^[^ ^35^, ^36^ ^]^ A high neutrophil‐to‐lymphocyte ratio, an inflammatory marker representing changes between inflammation and host immunity, correlates with worse overall survival in patients with PTCL.^[^ ^37^ ^]^ Moreover, autoregulatory cytokines, including IFN‐γ (secreted by CD4+ malignant T cells) and IL‐6 (secreted by activated granulocytes), could mediate granulocyte‐lymphoma interaction, thereby triggering inflammatory symptoms, enhancing lymphoma infiltration and affecting the survival of patients with PTCL.^[^ ^38^ ^]^ Similarly, increased serum IL‐10 levels predict poor survival and early recurrence in patients with PTCL.^[^ ^39^ ^]^ An elevated C‐reactive protein level, a crucial marker of inflammation, also has a poor independent prognostic value in patients with AITL.^[^ ^17^ ^]^ IL4 is an inflammatory cytokine primarily produced by type 2 helper T cells and a potent growth factor of B cells that drives helper T cell differentiation.^[^ ^40^ ^]^ In our study, IL4 was primarily expressed in T cells, especially in *cGAS+/STING*+ Tfh tumor cells in the RR group, and IL4R was predominantly expressed in T and B cells. The function of IL4/IL4R may be to accelerate AITL progression and trigger the transition from AITL to B cell lymphoma, indicating that IL4/IL4R is a potential pharmacological target. The cGAS‐STING pathway is closely associated with inflammation, a potential therapeutic target in inflammatory diseases,^[^ ^41^ ^]^ and could predict distinct prognoses in patients with different tumors.^[^ ^42^, ^43^, ^44^ ^]^ In our study, high cGAS‐STING pathway expression indicated a worse prognosis in patients with PTCL, which may further verify the prognostic value of inflammatory markers and partly unveil the underlying mechanism. The CSPS, defined by the seven most closely related marker genes, maybe a potential prognostic tool for PTCL.

Therapy‐induced DNA damage is a crucial source of cytosolic dsDNA in cancer cells, which activates the cGAS‐STING pathway. Under genotoxic stress, the cGAS‐STING pathway transmits nuclear DNA damage signals to the dynamic assembly of Hippo‐striatin‐interacting phosphatase and kinase (STRIPAK) via TBK1; subsequently, Hippo kinase is inactivated by STRIPAK, thereby increasing the DNA repair capacity of cancer cells and endowing these cells with chemotherapy resistance.^[^ ^45^ ^]^ In esophageal carcinoma, the Janus kinase‐2/STAT3 pathway, downstream of the cGAS‐STING pathway, is phosphorylated, enhancing the arrest of cell cycle‐mediated DNA repair and antagonizing the radiation‐induced killing effect.^[^ ^46^ ^]^ DOX‐induced cytoplasmic release of mitochondrial DNA activates the cGAS‐STING‐TBK1 pathway, triggering the expression of specific clusters of IFN‐stimulated genes, including poly (ADP‐ribose) polymerase 9. This gene expression enhances nuclear DNA repair, ultimately leading to DOX resistance.^[^ ^47^ ^]^ In our study, scRNA‐seq and bulk RNA sequencing revealed a positive correlation between cGAS‐STING and DNA repair pathways. G150, a human cGAS inhibitor, suppressed DNA repair in PTCL cells. DOX and ETO treatments induced cGAS‐STING pathway activation in residual PTCL cells, and G150 treatment improved chemotherapy efficacy. Overall, the cGAS‐STING pathway activation‐induced DNA repair may be the underlying mechanism of relapse and progression after chemotherapy in patients with PTCL.

Therapy‐induced DNA damage activates the cGAS‐STING pathway, ultimately inducing type I IFN response.^[^ ^48^, ^49^ ^]^ Activating a type I IFN response induces IFN‐γ generation by natural killer and T cells. This process enhances cytokine synthesis, such as CXCL10, fostering an improved immune response. Consequently, these cytokines facilitate the movement of circulating immune cells, particularly dendritic cells, B cells, CD4+ Th1 cells, CD8+ T effector cells, and tumor‐associated macrophages, to tumor cells.^[^ ^50^ ^]^ Activating this pathway results in immune cell infiltration into the tumor microenvironment. More importantly, the type I IFN response predominantly leads to upregulating immune checkpoint genes such as *PD‐1*, *CTLA‐4*, and *LAG3* in immune cells. Constant and chronic cGAS‐STING pathway activation may amplify this effect and induce an inhibitory inflammatory immune microenvironment. Simultaneously, activating the cGAS‐STING pathway can enhance DNA repair, leading to chemotherapy resistance. Therefore, high levels of inflammatory markers and high CSPS are prognostic markers, indicating poor outcomes in PTCL. However, the key to the transformation of the antitumor role of the cGAS‐STING pathway into a pro‐tumor effect is still unclear. Tumor genotype, chromosomal instability state, drug type, number of chemotherapy cycles, and many other factors may influence this process.

CLK1 can regulate the activity of splicing factors, further influencing cytosolic nascent RNA production. Single‐cell dynamic RNA sequencing is an efficient tool for detecting new RNAs.^[^ ^25^ ^]^ In our study, based on G150 exposure, adding the CLK1 inhibitor TG003 reduced proliferation‐ and DNA repair‐associated nascent RNAs, which may explain the synergy between TG003 and G150 in inhibiting PTCL cell proliferation. Promoting proliferation and DNA repair resulting from increased CLK1 expression may be the underlying mechanism of cGAS inhibitor antagonism. We verified the advantages of single‐cell dynamic RNA sequencing for exploring nascent RNAs and identified a combination regimen based on cGAS‐STING pathway inhibitors. To our knowledge, this is the first study to explore the function and possible mechanism of action of CLK1 in PTCL.

The novelty and advantages of this study are evident. This is the first study to report the pro‐tumor effects of the cGAS‐STING pathway in PTCL. Secondly, we used various innovative techniques, including single‐cell RNA sequencing, imaging mass cytometry, single‐cell dynamic RNA sequencing, and protein mass spectrometry, to ensure the credibility of our results. In addition to in vitro experiments, immunocompetent and immunodeficient mice were used to assess the antitumor effects of cGAS inhibitors, increasing their reliability for potential use in PTCL patients. Third, in addition to exploring the function and mechanism of the cGAS‐STING pathway, we found that cGAS inhibitors had a synergistic effect with chemotherapeutic agents and the CLK1 inhibitor TG003, providing a foundation for its clinical transformation. We believe our study contributes to a better understanding of the importance of the cGAS‐STING pathway in PTCL.

Our study had some limitations that should be addressed in future research. First, accounting for the high heterogeneity of PTCL even in the same subtype, especially in patients from different centers and regions, efforts are still needed for the clinical application of CSPS, including enlarging the cohort numbers in our centers and centers from other regions. Secondly, our study focused on the effect of the cGAS‐STING pathway on tumor cells; however, its influence on the tumor microenvironment in PTCL remains unclear. The relationship between these two effects in different cancers is expected to become a topic for future research. Third, although the cGAS‐STING pathway induced DNA repair in PTCL, the detailed mechanism remains unclear, as are the complex interactions among G150 treatment, increased CLK1 expression, and CLK1‐regulated splicing factor activity.

## 4 Conclusion

Our study shows that the cGAS‐STING pathway partially promotes PTCL progression by enhancing DNA repair and proliferation, which may also trigger chemotherapy resistance and relapse. Our study may partly reveal the deficiency of first‐line therapy and lay the foundation for the clinical application of cGAS‐STING pathway‐associated inhibitors in PTCL. Moreover, our findings imply the potential of anti‐inflammatory drugs for treating PTCL. Thus, CLK1 may be an effective tool for selecting patients eligible for cGAS inhibitor treatment. In addition, the CSPS optimized the performance of existing PTCL prognostic models. However, our study was limited by a small number of patients and PTCL subtypes. Therefore, international, multicenter, large‐scale clinical trials are required to validate our findings.

## 5 Experimental Section

### Cell Culture

Human PTCL cell lines (Hut78, MT‐4, and H9) and the TCL cell line (EL4) isolated from C57BL/6 mice were purchased from Nanjing Daona Biotechnology Limited Company (Nanjing, Jiangsu, China). Human PTCL cell lines (Karpas299 and Hut102) were purchased from KeyGEN BioTechnology Co Ltd. (Nanjing, Jiangsu, China). All cell lines were authenticated using short tandem repeat profile analysis. Cell lines were sustained for less than 6 months after resuscitation and were mycoplasma‐free. Cell lines were cultured in RPMI1640 medium (HyClone, Logan, UT, USA) with 10% fetal bovine serum (FBS; Bio‐channel, Nanjing, Jiangsu), streptomycin (50 U/mL), and penicillin (50 U/mL), at 37°C in a 5% CO_2_ atmosphere.

### Patients and Samples

This study was reviewed and approved by the Ethics Committee of Nanjing Medical University (approval no. 2023‐SRFA‐306). The study was performed after obtaining written informed consent from the patients following the principles outlined in the Declaration of Helsinki. For this study, 35 lymph node samples were obtained from patients who underwent lymphadenectomy for PTCL and were used for tissue microarray immunofluorescence at Jiangsu Province Hospital (Nanjing, Jiangsu, China). The personal data processing complied with Data Protection Laws. The diagnosis of PTCL was determined primarily according to the revised 4th edition of the WHO Classification of Haematolymphoid Tumors, and the diagnosis was reviewed and approved by two specialists in the Department of Pathology at Jiangsu Province Hospital.^[^ ^51^ ^]^ Clinic‐pathological stages of patients with PTCL were determined according to the 2014 Lugano revised edition of the Ann Arbor classification of malignant tumors.^[^ ^52^ ^]^ Tissue samples for single‐cell RNA sequencing were obtained from patients with AITL (*n =* 8), aged 43–78, or normal lymph nodes (*n =* 1) at Jiangsu Province Hospital between June 2019 and December 2020 and were stored at Singleron Biotechnologies (Nanjing, Jiangsu, China). 51 AITL samples from patients aged 26–82 years were examined, with a median follow‐up of 13 months after lymphadenectomy for overall survival, defined as the period from diagnosis to death or the most recent clinic visit. All patients with AITL (*n =* 51) received immunosuppressive treatment or were autoimmune disease‐free. Overall survival (OS) was set in the period from the date of diagnosis to death or to the most recent clinic visit. Relapsed AITL was defined as a complete response after first‐line treatment but relapse. Refractory AITL was defined as the failure to achieve a partial response after four cycles of standard first‐line treatment.

### Drugs

G150 (cat. no. HY‐128583; MedChemExpress, NJ, USA); H‐151 (cat. no. S6652, Selleck, Houston, TX, USA); TBK1/IKKε‐IN‐2 (cat. no. S0425, Selleck); Stattic (cat. no. S7024; Selleck); diABZI (cat. no. S8796; Selleck); G140 (cat. no. HY‐133916; MedChemExpress); RU.521 (cat. no. S6841; Selleck); TG003 (cat. no. S7320; Selleck), DOX (cat. no. S1208, Selleck); etoposide (cat. no. HY‐13629, MedChemExpress).

### Mice

Six‐week‐old C57BL/6 and NOD‐SCID mice were purchased from Gempharmatech Company (Shanghai, China). All mice were specific pathogen‐free and housed under high‐barrier conditions, according to the guidelines of the Jackson Laboratory, in the animal facility of the Animal Experimentation Center, Nanjing Agricultural University.

### scRNA‐seq and Analysis

Single‐cell suspensions were prepared from lymph nodes and loaded onto microfluidic devices. scRNA‐seq libraries were constructed and sequenced on an Illumina NovaSeq 6000 (Illumina, San Diego, CA, USA) with 150 bp paired‐end reads. Bioinformatics analysis included dimension reduction and clustering, new to total RNA calculation, metabolic labeling‐based RNA velocity analysis, pathway enrichment analysis, pseudo‐trajectory analysis, pySCENIC, and scRNA‐based copy number alteration detection. The details are described in Supplementary Information.

### Protein Mass Spectrometry

The peptide of two million cells was taken from each sample, and chromatographic separation was performed using a nanoliter flow rate Easy nLC 1200 chromatography system (Thermo Fisher Scientific, Waltham, MA, USA). Buffer: solution A was 0.1% formic acid in the water, and solution B was 80% ACN/0.1% formic acid. The column was equilibrated with a 100% liquid solution (A). After injection, the sample was separated using a gradient analysis column at a 300 nl min^−1^ flow rate. The liquid phase separation gradients were as follows: 0–3 min, B‐liquid linear gradient from 2–8%; 3–81 min, B‐liquid linear gradient from 8–40%; 81–83 min, B‐liquid linear gradient from 40–95%; and 83–90 min, solution B was maintained at 95%. Peptide isolation was followed by data‐dependent acquisition (DIA) mass spectrometry using a Q‐Exactive HF‐X mass spectrometer (Thermo Fisher Scientific). The analysis time was 90 min; detection mode: positive ion; precursor scan range: 390–1210 m/z; primary mass spectral resolution: 120 000; AGC target: 3e6; first‐stage Maximum IT 50 ms. DIA method acquisition: secondary mass spectral resolution: 30000; AGC target: 1e6; maximum IT: auto; loop count: 18; isolation window: 15 m/z; normalized collision energy: 28.

### Imaging Mass Cytometry Staining for Formalin‐Fixed Paraffin‐Embedded Sections

Formalin‐fixed paraffin‐embedded sections were preheated, dewaxed, dehydrated, and hydrated as previously described.^[^ ^53^ ^]^ Subsequently, antigen retrieval and blocking were performed. Sections were incubated with antibodies and stained with Intercalator‐Ir in Maxpar phosphate‐buffered saline (PBS). Finally, the sections were observed and photographed using a Hyperion Imaging System(Standard BioTools, San Francisco, CA, USA). The details are described in Supplementary Information.

### CCK8 Assay

Cell viability and proliferation were assessed using the CCK8 proliferation assay (cat. no. K1018; ApexBio Technology, Houston, TX, USA) after administering different drugs to PTCL cell lines.

### Flow Cytometry

Human and murine PTCL cells were harvested and mechanically dissociated into single‐cell suspensions to assess phenotypic markers. The cells were pelleted, resuspended in PBS, and incubated for 30 min at 4 °C with the antibodies listed in Table S3 (Supporting Information). The acquisition was performed using a Beckman Coulter instrument (RRID:SCR_008940; Brea, CA, USA), and the data were analyzed using FlowJo software (RRID:SCR_008520).

For the apoptosis assay, cells were washed twice with cold PBS and resuspended in a binding buffer. Apoptosis was quantified using flow cytometry (BD Biosciences, Franklin Lakes, NJ, USA) after 15 min of incubation at 4 °C with Annexin V and propidium iodide. Data analysis was performed using CytExpert FlowJo software (Version 10.4).

### Lentiviral Vectors Transfection

cGAS and CLK1 expression vectors were constructed. Vectors expressing cGAS transcript target sequences were: 5′‐Ccgg GCCTTCTTTCACGTATGTACC TTTTTg‐3′. Vectors expressing CLK1 transcript target sequences were: 5′‐Ccgg GGAATGGTTTGAGCATCATGG TTTTTg‐3′. cGAS expression was confirmed using RT‐PCR and WB. Lentiviral vector transfection was performed according to the manufacturer's instructions (GeneChem, Shanghai, China).

### Real‐Time Polymerase Chain Reaction (PCR)

RNA was extracted using TRIzol reagent (cat. no. 15596018; Ambion, Austin, TX, USA). Sample RNA content was measured using a NanoDrop 2000 (ThermoFisher Scientific, NewYork, NY, USA). HiScript III RT SuperMix for qPCR (+gDNA wiper) (Vazyme, Nanjing, Jiangsu, China) was used to reverse transcribe RNA into cDNA using GeneExplorer (Bioer, Hangzhou, Zhejiang, China). RT‐PCR was performed using 2×Taq Pro Universal SYBR qPCR Master Mix (Vazyme, Nanjing, Jiangsu, China) in a 10 µL template with a fluorescence qPCR detection system (Bioer). The primers used in this study are listed in **Table** **1**.

**Table 1:** The primer sequences used for reverse transcription PCR.

Primer	Forward (5′ to 3′)	Reverse (5′ to 3′)
CLK1	AGAGACCATGAAAGCCGGTAT	CATGTGAACGACGATGTGAAGT
CDK13	CTTCAGGTAACGAAGGTGGAAAA	CCTTGGCTCCTATGCTTGGTG
UIMC1(RAP80)	CCTTGGGACCACACTGAAAAA	GCATCTGCTAGGGTAGGTAGAC
GAPDH	GGAGCGAGATCCCTCCAAAAT	GGCTGTTGTCATACTTCTCATGG
XRCC4	ATGTTGGTGAACTGAGAAAAGCA	GCAATGGTGTCCAAGCAATAAC
ERCC2	GGAAGACAGTATCCCTGTTGGC	CAATCTCTGGCACAGTTCTTGA
TREX1	CGCATGGGCGTCAATGTTTT	GCAGTGATGCTATCCACACAGAA
XRCC5	GTGCGGTCGGGGAATAAGG	GGGGATTCTATACCAGGAATGGA
XRCC6	GTTGATGCCTCCAAGGCTATG	CCCCTTAAACTGGTCAAGCTCTA
RAD54B	GCCAAACACTGATGATTTGTGG	CCTGAGAAGAATGCGAGATAGC
ERCC4	GGAACTGCTCGACACTGACG	GCGAGGGAGGTGTTCAACTC
RAD23A	AGACGGTGAAGGTGCTAAAGG	TGATAGGGACATCGTCACTCAAG
ERCC6L	CTCTGGCTTGCTACTTTATCGAG	TGCATCAAACATACCGGAAAGG
BCCIP	TCAAGAGTTGGTTCTACGCTTC	CATGGGCAGAGCGATCTGT
BABAM2	TGGCCTTGAACCGAATATCTCC	GCATCCAGTCCCACTTTTCCAT

### Western Blotting

WB was performed according to standard procedures. The protein bands were quantified using the Fiji software (RRID: SCR_00228). All antibodies used for WB are listed in Table S3 (Supporting Information).

### Immunofluorescence Staining

Cell suspensions (2 × 10^4^ cells) were dropped onto a microscope slide (cat. no. 02286‐AB, SPI Supplies, West Chester, PA, USA), and the slide was incubated at 37 °C for ≈30 min until the cells formed films and attached to the slides. The cells were fixed with 4% paraformaldehyde and blocked with 0.5% TritonTMX‐100. Cells were stained with primary antibodies at 4 °C overnight, followed by incubation with fluorescently labeled secondary antibodies for 1 h at room temperature, protected from light. An anti‐fade solution containing 2‐(4‐amidinophenyl)−6‐indolecarbamidine dihydrochloride (DAPI) was added dropwise, and the coverslips were mounted. The cells were observed and photographed using a Stellaris STED laser confocal microscope (Stellaris STED, Leica, Germany) or a Thunder Imager Fast High‐Resolution Inverted Fluorescence Imaging System (Thunder DMi8, Leica, Germany).

### IHC and Analysis

IHC was performed according to standard procedures. The antibodies and reagents used for IHC are listed in Table S3 (Supporting Information). Expression of relevant indicators by neoplastic cells was evaluated using the following scores: 1) Ki67 was scored based on positive rate (the proportion of positive cells to total cells): <50%, 50–70%, and >70%; 2) Cleaved‐caspase3, γ‐H2AX, CLK1, p‐STING, dsDNA, cGAS, and STING were scored based on the immunoreactivity score (IRS): the staining degree (0–3 points) and positive rate (0–4 points) in the immunohistochemical section were scored and multiplied to obtain the comprehensive score (0–12 points). The degree of staining was scored according to the staining characteristics of the target cells as follows: 0, no staining; 1 point for light yellow; 2, brown; 3, brown. The positive rate was scored as follows: 0–5%, 0 points; 6–25%, 1 point; 26–50%, 2 points; 51–75%, 3 points; and >75%, 4 points. By multiplying the staining degree (0–3 points) and positivity rate (0–4 points), we obtained the IRS.

### Multiplex Immunofluorescence Staining

The tissue chip was incubated for 1 h at 63 °C and dewaxed in a fully automatic dyeing machine. The slides were then placed in a repair solution for antigen retrieval, followed by natural cooling. Commercial H_2_O_2_ was used to remove endogenous peroxidase, and a blocking buffer was added dropwise. The samples were incubated with primary and secondary antibodies at room temperature. The samples were stained with opal dye, followed by antibody removal. Other markers were stained before the anti‐fade solution containing DAPI was added, and the coverslips were mounted. Finally, as described above, the cells were observed and photographed for immunofluorescence staining.

### Comet Assay

The comet assay was performed using a Comet Assay Kit (SCGE) (cat. no. KGA240, KeyGEN BioTech, Nanjing, Jiangsu, China) according to the manufacturer's instructions. Briefly, cells embedded on slides in 0.7% low‐melting‐point agarose gels were lysed for 2 h and incubated in an alkaline electrophoresis buffer for 30 min before being electrophoresed for 30 min (25 V). The cells were neutralized and stained with propidium iodide before fluorescence microscopy imaging. For each sample, 100 cells were randomly selected to determine the diameter of nuclear DNA and the length of DNA migration, which were analyzed using the Comet Assay Software Project Laboratory (RRID:SCR_007249).

### In Vivo Experiment

A schematic illustration of the experimental design for establishing the tumor‐burden model followed by RU.521 treatment is shown in Figure 3I. Two groups of 6‐week‐old C57BL/6 mice (six mice in each group) were subcutaneously injected with 5×10^6^ EL4 cells to evaluate the impact of cGAS inhibition (RU.521) on murine T‐cell lymphoma. From the sixth day, one group received treatment with 15 mg k^−1^g RU.521, and the other group received PBS, all by intraperitoneal injection, every two days for 12 days.

Schematic illustrations of the experimental design for establishing the xenograft model, followed by treatment with different drugs, are shown in Figure 5L. Four groups (six mice in each group) of 6‐week‐old NOD‐SCID mice were subcutaneously injected with 1 × 10^7^ MT‐4 cells to study the effects of cGAS (G150) and CLK1 inhibition (TG003) in mice with human PTCL. On the ninth day, G150 was administered to the mice in the G150 monotherapy and combination therapy groups at 10 mg k^−1^g by intraperitoneal injection every two days. TG003 was administered at 50 mg k^−1^g by oral gavage to the mice in the TG003 monotherapy and combination therapy groups every day for 12 days.

Schematic illustrations of the experimental design for establishing the xenograft model, followed by treatment with different drugs, are shown in Figure 7O. 1×10^7^ MT‐4 cells were subcutaneously injected into three groups of 6‐week‐old NOD‐SCID mice (six mice in each group) to study the effects of cGAS inhibition (G150) and DOX in mice bearing human PTCL. From the sixth day, G150 (10 mg k^−1^g) was administered to mice in the G150 monotherapy and combination therapy groups by intraperitoneal injection every two days, whereas DOX (2 mg k^−1^g) was administered to mice in the DOX monotherapy and combination therapy groups by intraperitoneal injection every day for 12 days.

All three experiments measured tumor volume using calipers every three days. All mice were euthanized after 12 days of drug administration. The tumors were removed, measured, photographed, and used for histopathological analyses. An overall sample size of six mice per group allowed the detection of a statistically significant difference in tumor growth among the different groups (p‐value <0.05; GraphPad Prism 9.3, RRID: SCR_002798). All procedures involving mice were reviewed and approved by the Animal Research Ethics Board of the Nanjing Agricultural University (approval no. PZW2022044, PZW2021020).

### Statistical Analysis

Differences between groups were assessed using the Student's t‐test for in vitro and in vivo studies. For bioinformatics analysis, statistical analyses were performed as described above. Survival curves were constructed using the Kaplan–Meier method, and survival differences were analyzed using the log‐rank test with a median follow‐up of 13 months. Gene co‐occurrence analysis was performed using Fisher's exact probability test. Spearman's correlation coefficient was used to exclude correlations between gene expression and patient age or Ann Arbor stage. The sample sizes (n) for each statistical analysis were included in the figure legends. Asterisks represent the p‐values: *p<0.05, **p<0.01, ***p<0.001, ****p<0.0001, ns: not significant. All statistical analyses were performed using GraphPad Prism 9.3 and R software on R Studio.

## Conflict of Interest

The authors declare no competing interests.

## Author Contributions

X.L., S.W., X.H., and X.C. contributed equally to this work. H.J., J.L., W.S., and L.F. designed and supervised the study. X.L., S.W., X.H., and X.C. performed the experiments and collected the data. X.Z. and W.Z. contributed to the in vivo experiments. X.L. and R.G. performed the immunostaining. S.W. and L.C. contributed to data interpretation. M.Z., Q.H., X.L., X.H., and Z.W. supported the data analysis. S.W. and X.L. analyzed the data and wrote the manuscript.

## Supporting information

Supporting Information

Supplemental Table 1

Supplemental Table 2

Supplemental Table 3

## Data Availability

The data that support the findings of this study are available from the corresponding author upon reasonable request.
