# Supplementary material for: Targeting the cGAS‐STING Pathway Inhibits Peripheral T‐cell Lymphoma Progression and Enhances the Chemotherapeutic Efficacy
Source: Adv Sci (Weinh). 2023 Dec 25;11(10):2306092. doi: 10.1002/advs.202306092 (PMC10933671; doi:10.1002/advs.202306092)
Supplement: Supplementary file 1 — Supporting Information [file ADVS-11-2306092-s004.pdf]

ADVANCED SCIENCE
Open Access

Supporting Information

for *Adv. Sci.*, DOI 10.1002/advs.202306092

Targeting the cGAS-STING Pathway Inhibits Peripheral T-cell Lymphoma Progression and Enhances the Chemotherapeutic Efficacy

Xueying Lu, Shunan Wang, Xin Hua, Xiao Chen, Mengtao Zhan, Qiaoyun Hu, Lei Cao, Zijuan Wu, Wei Zhang, Xiaoling Zuo, Renfu Gui, Lei Fan, Jianyong Li*, Wenyu Shi* and Hui Jin**

Supporting Information

Targeting the cGAS-STING pathway inhibits the progression and enhances the chemotherapy efficacy of Peripheral T-cell lymphoma

Xueying Lu, Shunan Wang, Xin Hua, Xiao Chen, Mengtao Zhan, Qiaoyun Hu, Lei Cao, Zijuan Wu, Wei Zhang, Xiaoling Zuo, Renfu Gui, Lei Fan, Jianyong Li,* Wenyu Shi,* and Hui Jin**

1. Supplementary Experimental Section Methods

Single-cell RNA sequencing and analysis: Tissue dissociation and single-cell suspension preparation: The lymph nodes samples were conserved in the GEXSCOPE Tissue Preservation Solution (Singleron, Nanjing, Jiangsu, China) and shipped to the Singleron lab (Nanjing, Jiangsu, China) with ice pack. The specimens were washed 3 times with Hanks Balanced Salt Solution (HBSS, Cat. No.14025-076, Gibco, USA) and shred into 1-2 mm pieces. Then the tissue debris were submitted to the digestion with 2 ml GEXSCOPE Tissue Dissociation Solution (Singleron, Nanjing, Jiangsu, China) at 37℃ for 15 min in 15 ml centrifuge tube (Falcon, Cat. No.352095, Germany) with sustained agitation. Cells were filtered through 40-micron sterile strainers (Falcon, Cat. No.352340, Germany) and centrifuged (eppendorf, 5810R) at 300g for 5 min. Then the supernatant was removed, and the pellets were resuspended in 1 ml PBS (Hyclone, Cat. No.SA30256.01, USA). To remove the red blood cells, which were frequently a significant portion of the cells produced, 2 ml RBC lysis buffer (Roche, Cat. No. 11814389001, Switzerland) was added to the cell suspension according to the manufacturer's protocol. Centrifuge the cells at 500g for 5 min in a microfuge at 15-25℃ and resuspend in PBS (Hyclone, Cat. No.SA30256.01, USA). The sample from the cell mixture was stained with trypan blue (Bio-RAD, Cat. No.#1450013, USA) and microscopically (Nikon, ECLIPSE Ts2, Japan) cell count to make the concentration was 1×10^5 cells/ml, and once the cell viability exceeded 80%, subsequent sample processing could be performed.

Metabolic labeling for Single-cell dynamic RNA sequencing: take out the labeling reagent (Singleron Biotechnologies, Nanjing, China) from -20℃ to thaw at room temperature, vortex for more than 10s, mix thoroughly and set it on ice. Add labeling reagent S^4U to the medium

according to 1:100 (Labeling reagent: medium) and mix thoroughly. The medium containing labeling reagent was added to the culture system treated with the drug or DMSO for 18 h, and continued to be incubated for 6 h at 37°C protected from light. After the end of cultivation, centrifuge the cell suspension at 300g for 5 min, remove the supernatant, resuspend the cells with PBS (HyClone, USA) and perform cell counting.

Reverse transcription-Polymerase Chain Reaction (RT-PCR), Amplification and Library Construction: Single-cell suspensions (2×10^5 cells/ml) with PBS (HyClone, Logan, UT, USA) were loaded onto microwell chip using the Singleron Matrix® Single Cell Processing System (Singleron Biotechnologies, Nanjing, China). Barcoding beads are subsequently collected from the microwell chip, followed by reverse transcription of the mRNA captured by the barcoding beads to obtain cDNA and PCR amplification. The amplified cDNA is then fragmented and ligated with sequencing adapters. The scRNA-seq libraries were constructed according to the protocol of the GEXSCOPE® Single Cell RNA Library Kits (Singleron Biotechnologies, Nanjing, China).[1] Individual libraries were diluted to 4 nM, pooled, and sequenced on Illumina novaseq 6000 with 150 bp paired end reads.

Primary analysis of raw read data: Raw reads from scRNA-seq were processed to generate gene expression matrixes using CeleScope (https://github.com/singleron-RD/CeleScope) v1.9.0 pipeline. Briefly, raw reads were first processed with CeleScope to remove low quality reads and with Cutadapt v1.17 to trim poly-A tail and adapter sequences.[2] Cell barcode and UMI were extracted. After that, STAR v2.6.1a were used to map reads to the reference genome GRCm38 (ensembl version 92 annotation).[3] UMI counts and gene counts of each cell were acquired with featureCounts v2.0.1 software, and used to generate expression matrix files for subsequent analysis.[4]

Quality control, dimension-reduction and clustering: The raw expression matrices of new and old UMI counts were summed up as total UMI counts and loaded into Seurat. Cells were filtered by gene counts below 200 and the top 2% gene counts and the top 2% UMI counts. Cells with over 20% mitochondrial content were removed. After filtering, qualified cells were retained for the downstream analyses. We used functions from Seurat (v3.1.2) for dimension-reduction and clustering. Then we used NormalizeData and ScaleData functions to normalize and scale all gene expression, and selected the top 2000 variable genes with FindVariableFeautres function for PCA analysis. Using the top 20 principle components, we separated cells into multiple clusters with FindClusters. Batch effect between samples was removed by Harmony. Finally, UMAP algorithm was applied to visualize cells in a two-dimensional space. Matrices of new, old and total transcripts were used in further analysis.

New to total RNA calculation: NTR (new to total RNA) was calculated as proportion of newly synthesized transcripts in whole transcripts based on UMI counts in each gene or cell. The result was projected to the UMAP/tSNE plot from Seurat clustering analysis for visualization consistency. NTR was also shown by Violin plot with statistical test.

Metabolic labeling-based RNA velocity analysis: Metabolic labeling-based RNA velocity analysis was performed to study transcriptional dynamics by scVelo (v.0.2.4) in Python. RNA Velocity was calculated using new/total RNAs as unspliced/spliced RNAs. The result was projected to the UMAP plot from Seurat clustering analysis for visualization consistency.

Pathway enrichment analysis: To investigate the potential functions, Gene Ontology (GO) and Kyoto Encyclopedia of Genes and Genomes (KEGG) analysis were used with the "clusterProfiler" R package (v3.16.1). Pathways with p value less than 0.05 were considered as significantly enriched. Selected significant pathways were plotted as bar plots. For GSVA pathway enrichment analysis, the average gene expression of each sample was used as input data. Gene Ontology gene sets including molecular function (MF), biological process (BP), and cellular component (CC) categories were used as reference.

Pseudotime Trajectory Analysis: monocle2: Cell differentiation trajectory was reconstructed with the Monocle2 (v2.10.0). For constructing the trajectory, top 2000 highly variable genes were selected by Seurat (v3.1.2) Find Variable Features, and dimension-reduction was performed by DDR Tree. The trajectory was visualized by plot cell trajectory function in Monocle2.

Transcription factor regulatory network analysis (pySCENIC): Transcription factor network was constructed by pyscenic (v0.11.0) using scRNA expression matrix and transcription factors (TF) in AnimalTFDB. First, GRNBoost2 predicted a regulatory network based on the co-expression of regulators and targets. CisTarget was then applied to exclude indirect targets and to search transcription factor binding motifs. After that, AUCell was used for regulon activity quantification for each cell. Cluster-specific TF regulons were identified according to Regulon Specificity Scores (RSS) and the activity of these TF regulons were visualized in heatmaps.

scRNA-seq based CNA detection: The InferCNA package was used to detect the CNAs. Genes expressed in more than 20 cells were sorted based on their loci on each chromosome. The relative expression values were centered to 1, using 1.5 standard deviation from the residual-normalized expression values as the floor and ceiling. A slide window size of 101 genes was used to smoothen the relative expression on each chromosome, to remove the

effect of genespecific expression. The CNA score of each cell was calculated as quadratic sum of CNAregon.

Bulk-RNA sequencing analysis: PTCL expression datasets were downloaded from gene expression omnibus (GEO) with accession ID GSE160119 and GSE51521. (1) Differential expression analysis: Differential expression analysis of two conditions/groups (two biological replicates per condition) was performed in R using with “limma” (v3.50.1) package. “limma” fits a linear model using weighted least squares for each gene and computes differential expression by empirical Bayes moderation of the standard errors towards a global value. The resulting P-values were adjusted using the Benjamini and Hochberg’s approach for controlling the false discovery rate. Genes with an adjusted P-value <0.05 were assigned as differentially expressed. (2) GO and KEGG enrichment analysis of differentially expressed genes: Gene Ontology (GO) enrichment analysis of differentially expressed genes was implemented by the cluster Profiler (v4.0.2) R package (https://guangchuangyu.github.io/software/clusterProfiler/), in which gene length bias was corrected. GO terms with corrected P value less than 0.05 were considered significantly enriched by differential expressed genes. KEGG is a database resource for understanding high-level functions and utilities of the biological system, such as the cell, the organism and the ecosystem, from molecular level information, especially large-scale molecular datasets generated by genome sequencing and other high-through put experimental technologies (http://www.genome.jp/kegg/). We used cluster Profiler R package to test the statistical enrichment of differential expression genes in KEGG pathways.

Imaging mass cytometry staining for FFPE sections: Bake the slides for 2 h at 60 °C in the slide oven/dryer. Alternatively, if no oven is available, a heat block set at 60 °C can be used. In either case, ensure that all visible wax is removed. Turn the heating block on to 96°C during the baking step. Prior to dewaxing, prepare 50 ml conical tubes containing 40 ml of antigen retrieval solution (10× diluted to 1×) and put the tubes in the heating block (96°C) with loose lids. Dewax the slides in fresh xylene in the fume hood for 20 min with loose lids. Hydrate the slides in descending grades of ethanol (100%, 95%, 80%, 70%), 5 min each. Wash the slides in Maxpar Water for 5 min in a Coplin jar placed on an orbital shaker plate with gentle agitation. Insert the slides with tissues into preheated antigen retrieval solution and incubate them for 30 min, leaving the lids loose. Following incubation, remove the slides from the heating block, place the tubes containing the antigen retrieval solution and slides on a lab bench, and cool them to 70°C by monitoring the temperature of the antigen retrieval solution for about 10 min. Wash the slides with Maxpar Water for 10 min in a Coplin jar with

gentle agitation (orbital shaker). Wash the slides with Maxpar PBS for 10 min with gentle agitation. Use a PAP pen to encircle the sample. Block with 3% BSA in Maxpar PBS for 45 min at room temperature in a hydration chamber. To prepare the antibody cocktail, calculate the total volume of antibodies at concentrations specific for the assay and bring the volume up to a final volume of 0.5% BSA in Maxpar PBS. Place the slides in a hydration chamber and pipette the antibody master mix onto the section. Incubate overnight with the antibody cocktail at 4°C in a hydration chamber. Wash the slides in 0.2% Triton X-100 in Maxpar PBS for 8 min with slow agitation in Coplin jars. Repeat. Wash the slides in Maxpar PBS for 8 min with gentle agitation. Repeat the washing. Stain the tissue with Intercalator-Ir in Maxpar PBS (300–500 μl/section for a 20 mm^2 section of 1:400 solution) for 30 min at room temperature in a hydration chamber. Wash the slides in Maxpar Water for 5 min with gentle agitation. Air-dry the slides for at least 20 min at room temperature. Observe and photograph with Hyperion Imaging System.

References

[1] Burak Dura, Jin-Young Choi, Kerou Zhang, William Damsky, Durga Thakral, Marcus Bosenberg, Joe Craft, Rong Fan, *Nucleic Acids Res*. **2019**, *47*, e16.

[2] M. Martin, *Embnet Journal* **2011**, *17*.

[3] A. Dobin, C. A. Davis, F. Schlesinger, J. Drenkow, C. Zaleski, S. Jha, P. Batut, M. Chaisson, T. R. Gingeras, *Bioinformatics* **2013**, *29*, 15.

[4] Y. Liao, G. K. Smyth, W. Shi, *Bioinformatics* **2014**, *30*, 923.

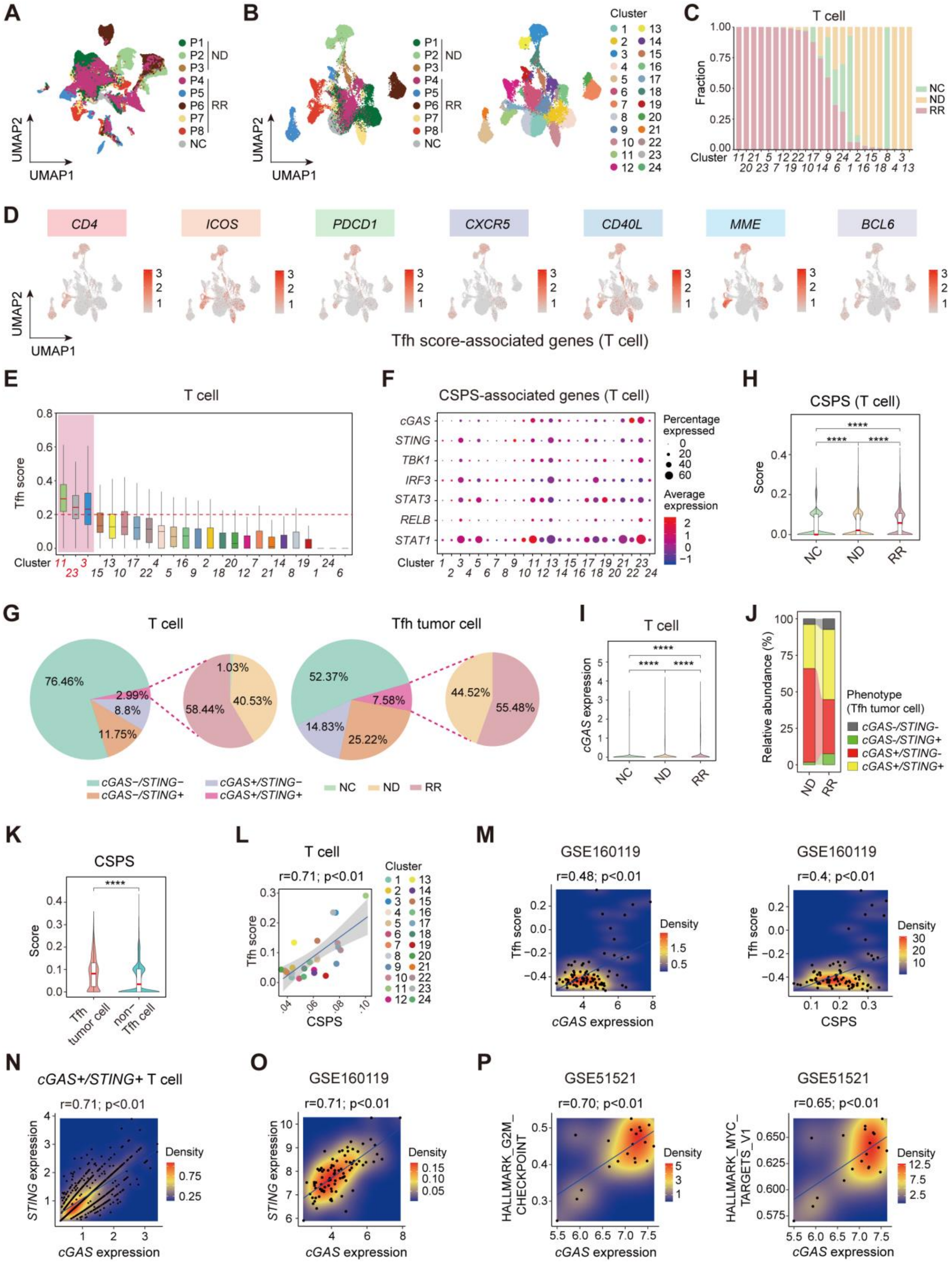
A
B
C
T cell
Cluster
Fraction
NC
ND
RR
UMAP1
UMAP2
D
CD4
ICOS
PDCD1
CXCR5
CD40L
MME
BCL6
Tfh score-associated genes (T cell)
E
T cell
Tfh score
F
CSPS-associated genes (T cell)
cGAS
STING
TBK1
IRF3
STAT3
RELB
STAT1
Percentage expressed
Average expression
H
CSPS (T cell)
Score
G
T cell
Tfh tumor cell
76.46%
2.99%
8.8%
11.75%
1.03%
58.44%
40.53%
52.37%
7.58%
14.83%
25.22%
44.52%
55.48%
cGAS−/STING−
cGAS−/STING+
cGAS+/STING−
cGAS+/STING+
I
T cell
cGAS expression
J
Relative abundance (%)
Phenotype (Tfh tumor cell)
K
CSPS
Tfh tumor cell
non-Tfh cell
L
T cell
r=0.71; p<0.01
CSPS
M
GSE160119
r=0.48; p<0.01
r=0.4; p<0.01
Density
N
cGAS+/STING+ T cell
r=0.71; p<0.01
STING expression
O
GSE160119
r=0.71; p<0.01
P
GSE51521
r=0.70; p<0.01
HALLMARK_G2M_CHECKPOINT
r=0.65; p<0.01
HALLMARK_MYC_TARGETS_V1

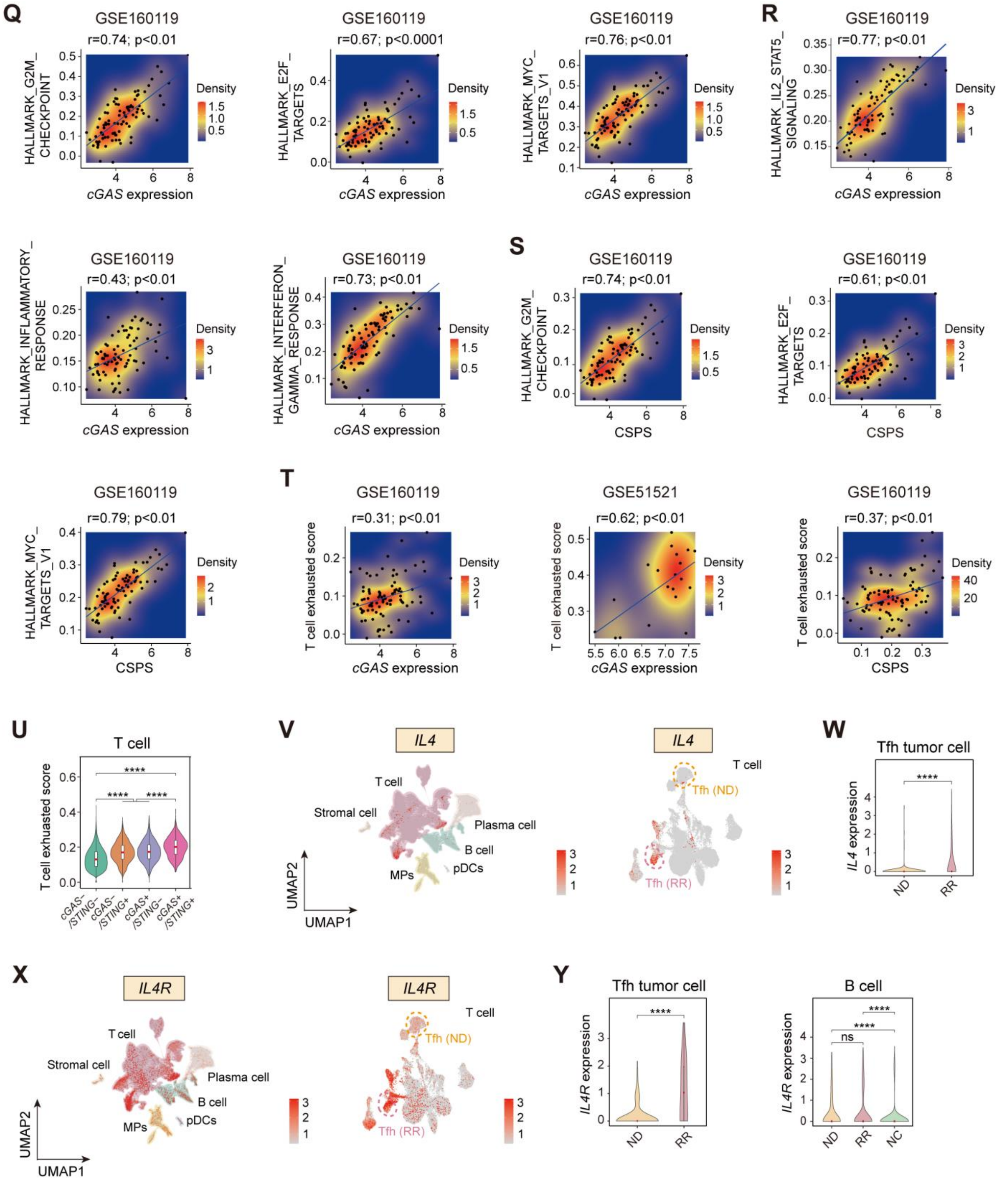

Figure S1. Expression and function of the cGAS-STING pathway in AITL detected by single-cell RNA sequencing. A) UMAP plot of 859,028 cells showing the patients in different groups. B) UMAP plots of 34773 T cells showing the patients in different groups (left) and the 24 clusters (right). C) Histogram showing the proportion of three groups (NC, ND, and RR) in 24 T cell clusters. D) UMAP plots showing the expression of seven Tfh score-associated genes (*CD4*, *ICOS*, *PDCD1*, *CXCR5*, *CD40L*, *MME*, and *BCL6*) in T cells. E) Box plot showing the Tfh score of 24 T cell clusters. F) Dot plot showing the expression of CSPS-

associated genes (*cGAS, STING, TBK1, IRF3, STAT3, RELB, STAT1*) in 24 T cell clusters. G) Pie charts showing the proportion of four phenotypes (*cGAS+/STING+*, *cGAS+/STING-*, *cGAS-/STING+*, and *cGAS-/STING-*) and the corresponding proportion of three groups (NC, ND, and RR) in *cGAS+/STING+* phenotype in T (left) and Tfh tumor cells (right). H) Violin plot showing the CSPS of T cells in three groups (NC, ND, and RR). I) Violin plot showing the *cGAS* expression in T cells of three groups (NC, ND, and RR). J) Alluvial plot showing the proportion of four phenotypes in Tfh tumor cells of ND/RR patients with AITL using imaging mass cytometry. K) Violin plot showing the CSPS in Tfh tumor cells and non-Tfh cells. L) Scatterplot showing the correlation between CSPS and Tfh score in 24 T cell clusters. M) Scatterplots with density showing the correlations between *cGAS* expression/CSPS and Tfh score in bulk RNA sequencing data of PTCL (GSE160119). N) Scatterplot with density showing the correlation between *cGAS* and *STING* expression in *cGAS+/STING+* T cells in single-cell RNA sequencing of AITL. O) Scatterplot with density showing the correlation between *cGAS* and *STING* expression in bulk RNA sequencing data of PTCL (GSE160119). P, Q) Scatterplots with density showing the correlations between *cGAS* expression and proliferation-associated pathways, including the G2M checkpoint, E2F targets, and MYC targets v1 pathways in bulk RNA sequencing data of AITL (GSE51521) (P) and PTCL (GSE160119) (Q). R) Scatterplots with density showing the correlations between cGAS expression and inflammation-associated pathways, including the IL2-STAT5 signaling, inflammatory response, and IFN-γ response pathways in bulk RNA sequencing data of PTCL (GSE160119). S) Scatterplots with density showing the correlations between CSPS and proliferation-associated pathways, including the G2M checkpoint pathway, E2F targets pathway and MYC targets v1 pathway in bulk RNA sequencing data of PTCL (GSE160119). T) Scatterplots with density showing the correlations between *cGAS* expression/CSPS and T cell exhausted score in bulk RNA sequencing data of AITL (GSE51521) and PTCL (GSE160119). U) Violin plot showing the T cell exhausted score in four phenotypes (*cGAS+/STING+*, *cGAS+/STING-*, *cGAS-/STING+*, and *cGAS-/STING-*) of T cells with scRNA-seq data. V) UMAP plots showing *IL4* expression in all cell types (left) and T cells (right). W) Violin plot showing the *IL4* expression in Tfh tumor cells of ND and RR groups. X) UMAP plots showing the expression of *IL4R* in all cell types (left) and T cells (right). Y) Violin plots showing the *IL4R* expression in Tfh tumor cells of ND/RR groups (left) and B cells of ND/RR/NC groups (right). ********$p<0.0001$, ns: not significant. The Wilcoxon test was used to detect differences between groups (R language). UMAP, Uniform Manifold Approximation and Projection; ND, newly diagnosed; RR, relapse or refractory; NC, normal

lymph node; Tfh, T follicular helper; CSPS, cGAS-STING pathway score; IL4, interleukin-4; IL4R, IL4 receptor

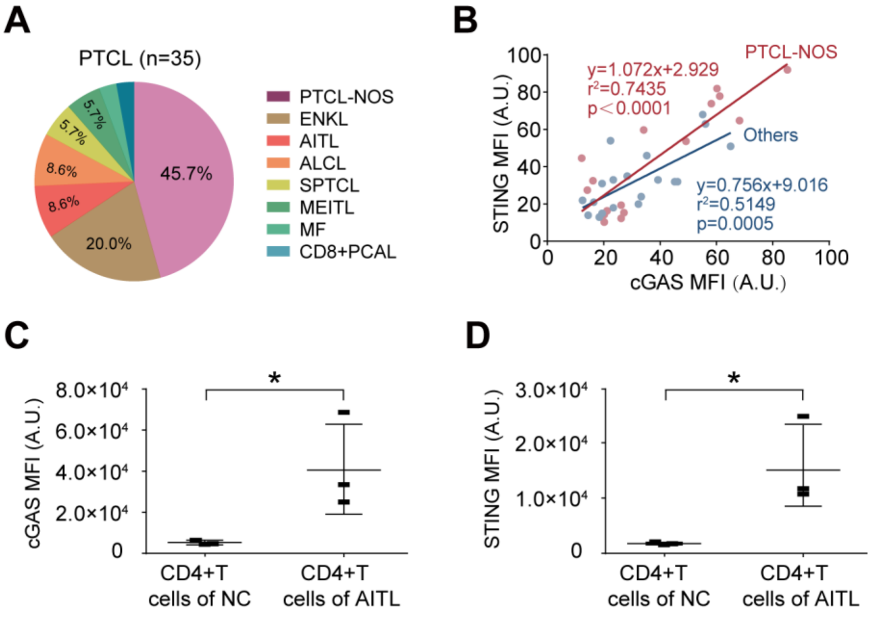

Figure S2. cGAS/STING is overexpressed in PTCL with high heterogeneity and could be used as a prognostic marker. A) Pie chart showing the proportion of eight different PTCL subtypes. B) Scatterplot showing the correlation between cGAS and STING expression with MFI in PTCL-NOS (red) and other PTCL samples (blue). C) cGAS expression in CD4+ T cells sorted from three normal lymph nodes (NC) and three AITL samples using flow cytometry (NC [n = 3], AITL [n = 3]). D) STING expression in CD4+ T cells sorted from three normal lymph nodes (NC) and three AITL samples using flow cytometry (NC [n = 3], AITL [n = 3]). *****$p<0.05$. Student's t-test was used to detect differences between groups (Software GraphPad Prism 9.3). PTCL, peripheral T-cell lymphoma; PTCL-NOS, PTCL-not otherwise specified; AITL, angioimmunoblastic T-cell lymphoma; ENKL, extranodal NK/T-cell lymphoma; ALCL, anaplastic lymphoma kinase positive anaplastic large cell lymphoma; MEITL, monomorphic epitheliotropic T-cell lymphoma; MF, mycosis fungoides; SPTCL, subcutaneous panniculitic T-cell lymphoma; PCAL, primary cutaneous acral lymphoma; MFI, mean fluorescence intensity; A.U, arbitrary unit; cGAS, cyclic GMP-AMP synthase; STING, stimulator of interferon genes; NC, normal lymph node

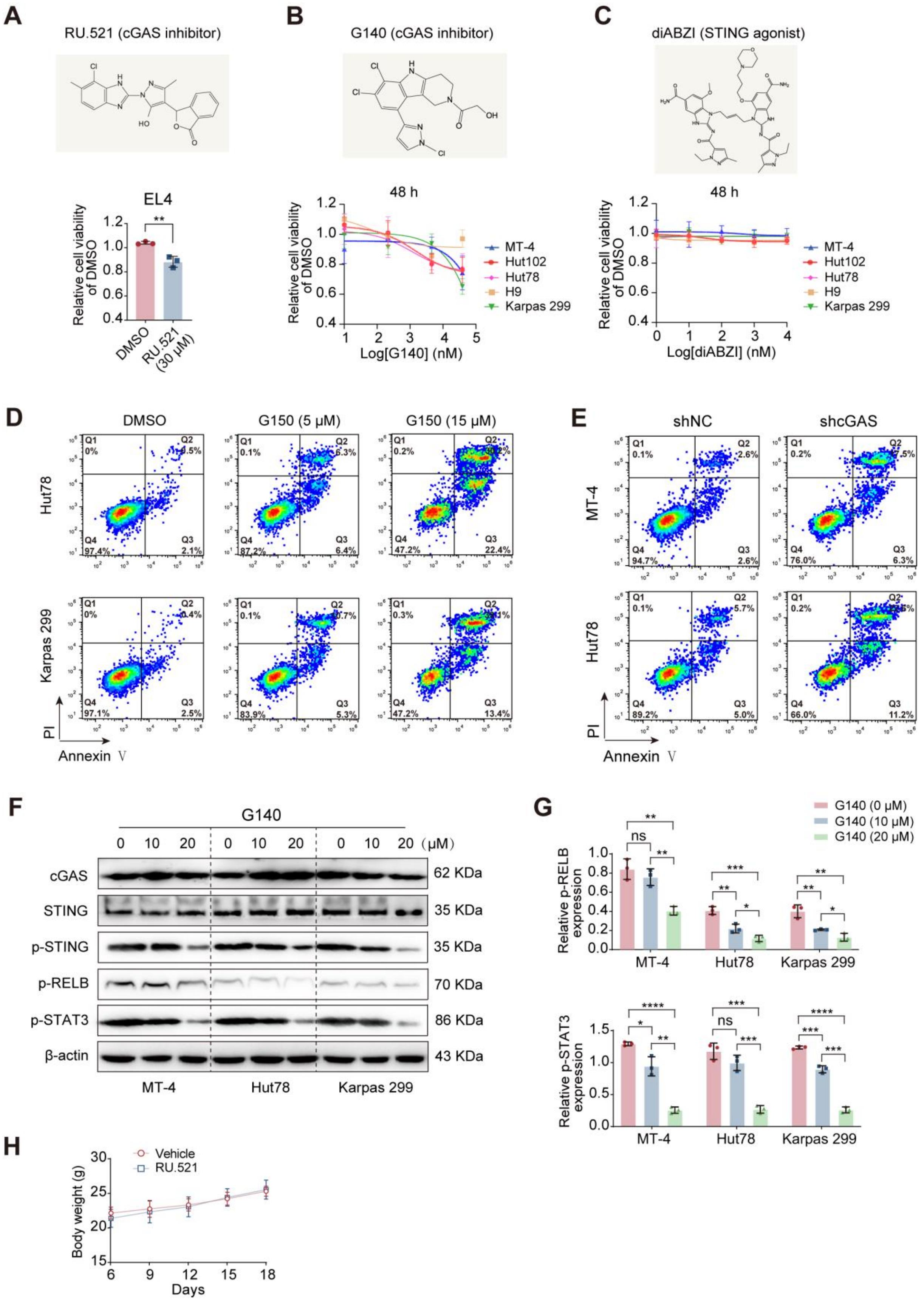

Figure S3. Targeting the cGAS-STING pathway inhibits PTCL proliferation and induces apoptosis. A) The CCK8 assay results showing the proliferation level of murine TCL cell line (EL4) treated with RU.521 for 48 h. B) The CCK8 assay results showing the proliferation

levels of PTCL cell lines (MT4, Hut102, H9, Hut78, and Karpas 299) treated with an cGAS inhibitor (G140) for 48 h. C) The CCK8 assay results showing the proliferation levels of PTCL cell lines (MT4, Hut102, H9, Hut78, and Karpas 299) treated with a STING agonist (diABZI) for 48 h. D) Flow cytometry results showing the apoptosis levels of PTCL cell lines (Hut78 and Karpas 299) treated with G150 or DMSO for 48 h. E) Flow cytometry results showing the apoptosis levels of two shcGAS or shNC PTCL cell lines (Hut78 and MT-4). F) WB results showing the expression of cGAS-STING pathway-associated protein in three PTCL cell lines (MT-4, Hut78, and Karpas 299) treated with G140 for 48 h. G) Quantification analyses showing p-RELB and p-STAT3 expression in (F). H) Body weight change of mice in the RU.521 administration and control groups (n=6). In vitro experiments were conducted in triplicate. *p<0.05, **p<0.01, ***p<0.001, ****p<0.0001, ns: not significant. Student's t-test was used to detect differences between groups (Software GraphPad Prism 9.3). cGAS, cyclic GMP-AMP synthase; STING, stimulator of interferon genes; DMSO, dimethyl sulfoxide; PI, propidium iodide; shNC, knocking down nothing by shRNA; shcGAS, knock out cGAS by shRNA; p-STING, phosphorylated STING; p-TBK1, phosphorylated TBK1; p-IRF3, phosphorylated interferon regulatory factor 3; p-RELB, phosphorylated RELB; p-STAT3, phosphorylated STAT3; WB, western blotting

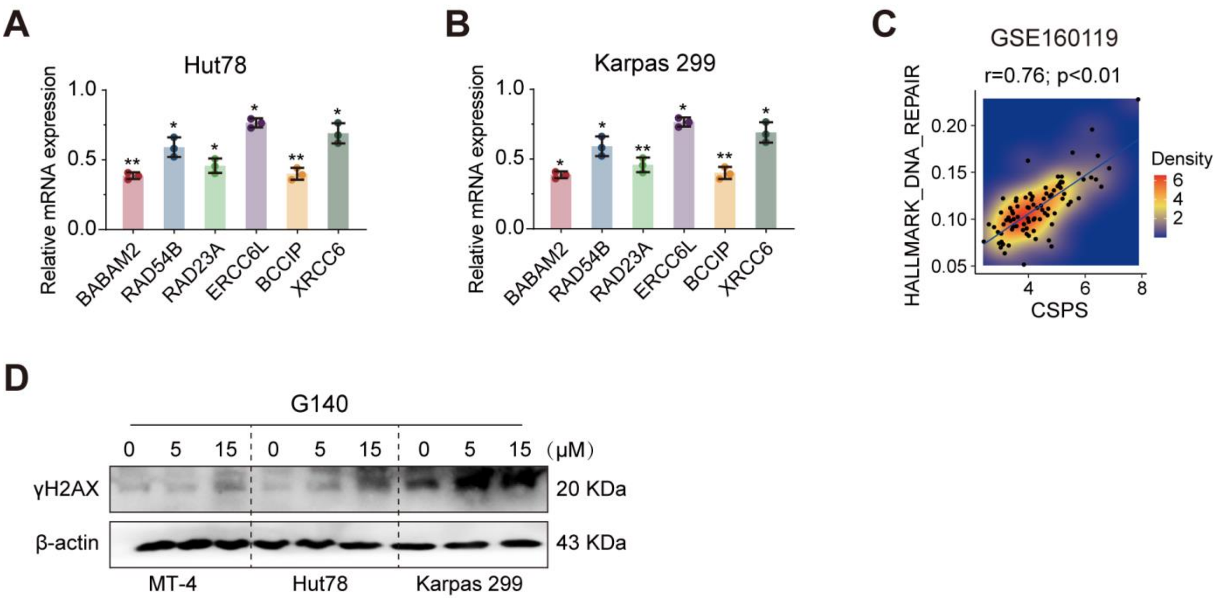

Figure S4. Targeting cGAS attenuates DNA damage repair. A, B) The qPCR results showing the downregulation of the six DNA repair-related genes in PTCL cell lines (Hut78 and Karpas 299) treated with G150 for 48 h. C) Scatterplot with density showing the correlation between CSPS and DNA repair pathway in bulk RNA sequencing data of PTCL (GSE160119). D) WB results showing γH2AX expression in PTCL cell lines (MT-4, Hut78, and Karpas 299) treated

with G140 for 48 h. Experiments were conducted in triplicate. Student's t-test was used to detect differences between groups (Software GraphPad Prism 9.3). *p<0.05, **p<0.01. CSPS, cGAS-STING pathway score; WB, western blotting

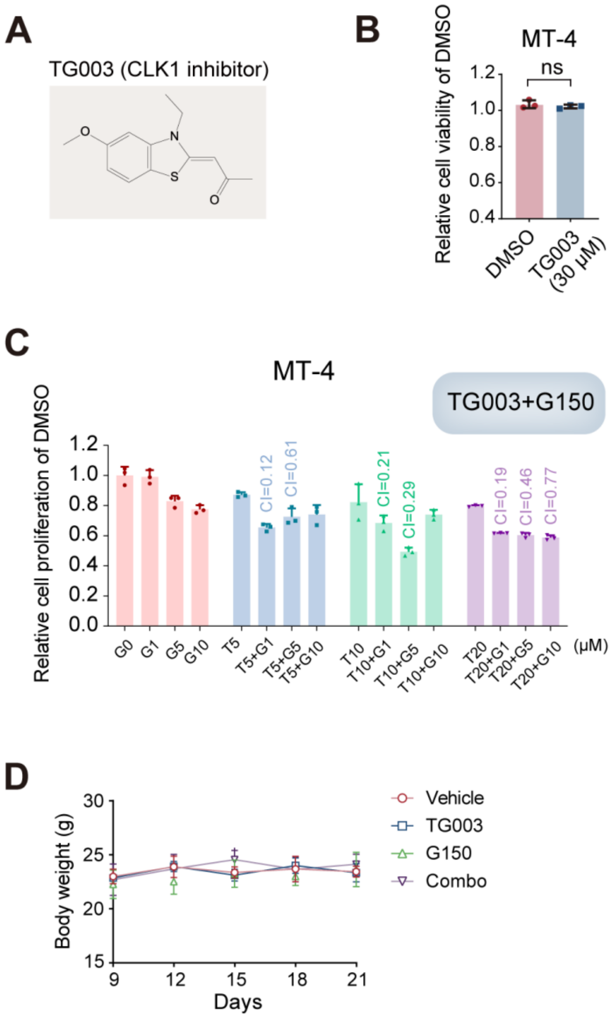

Figure S5. CLK1 may be a potential sensitivity indicator of cGAS inhibitor G150 and is a promising target for combination therapy. A) Chemical formula of TG003. B) CCK8 assay results showing the proliferation level of MT-4 cells treated with TG003 for 48 h. C) CCK8 assay results show the relative proliferation of the PTCL cell line treated by G150 or G150 plus TG003 combination treatment for 48 h, with the CI. D) Body weight change of mice in four groups during drug administration (n = 6). In vitro experiments were conducted in triplicate. Ns: not significant. Student's t-test was used to detect differences between groups (GraphPad Prism 9.3). CLK1, Cdc2-like kinase 1; DMSO, dimethyl sulfoxide; CI, combination index

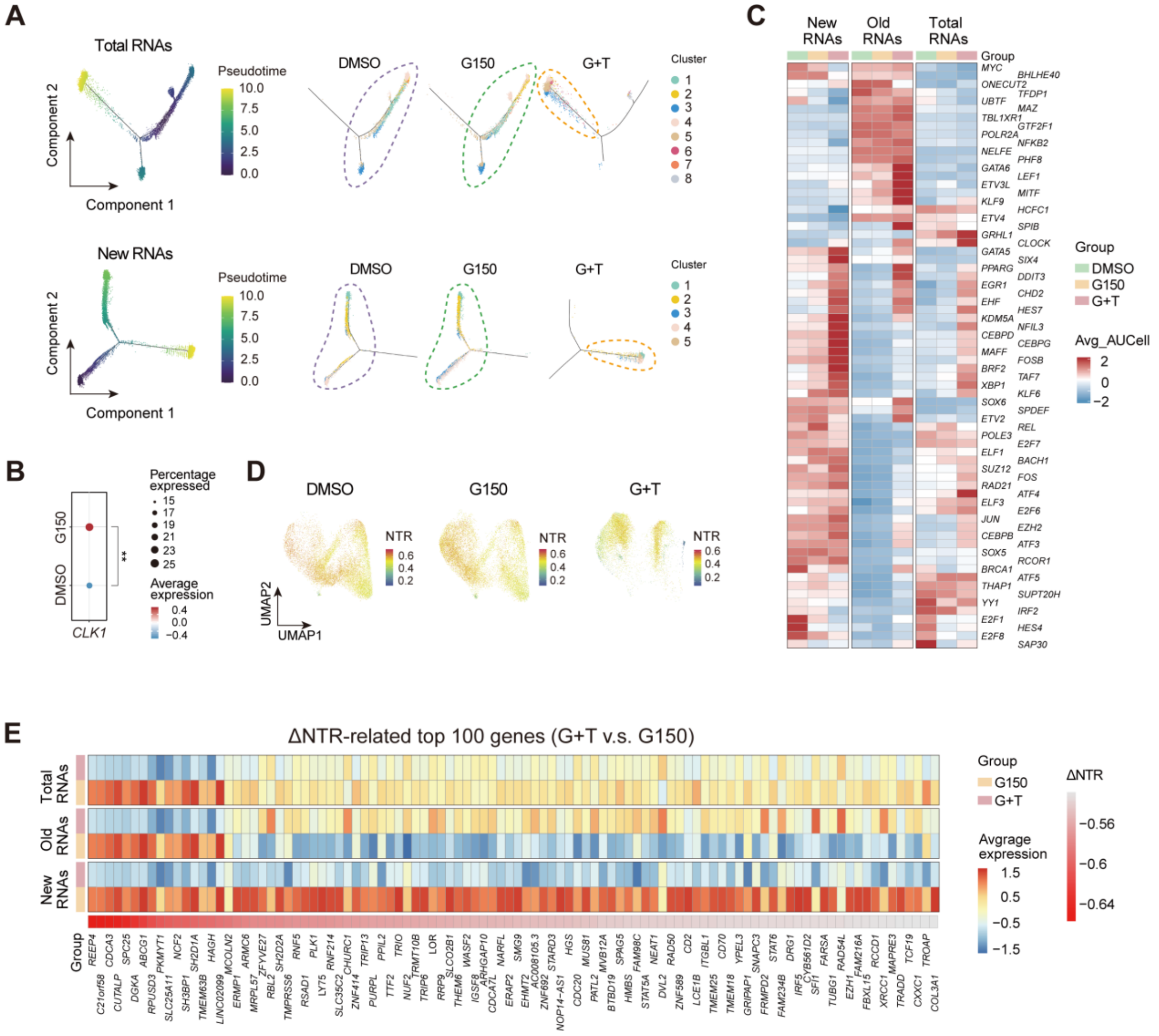

Figure S6. Single-cell dynamic RNA sequencing reveals that TG003 functions by inducing reduced proliferation-related nascent RNAs based on G150 treatment. A) Pseudotime analysis of trajectory according to total (upper) and new RNAs (below) in three groups (DMSO, G150, and G150 plus TG003 group). B) Dot plot showing the *CLK1* expression in the DMSO and G150 groups according to total RNAs. C) The heatmap of the selected transcription factors in three groups (DMSO, G150, and G150 plus TG003 group) according to new, old or total RNAs. D) UMAP plots separately showing the NTR of three groups (DMSO, G150, and G150 plus TG003 group). DMSO (n = 1), G150 (n = 1), G150+TG003 (n = 1). E) Heatmap showing the expression of ΔNTR-related top 100 genes according to new RNAs between the G150 and combination groups (G+T) and their expression in old or total RNAs. **$p<0.01$. The Wilcoxon test was used to detect differences between groups (R language). DMSO, dimethyl sulfoxide; G+T, G150 plus TG003; CLK1, Cdc2-like kinase 1; NTR, new-to-total RNA ratio; ΔNTR, NTR difference value

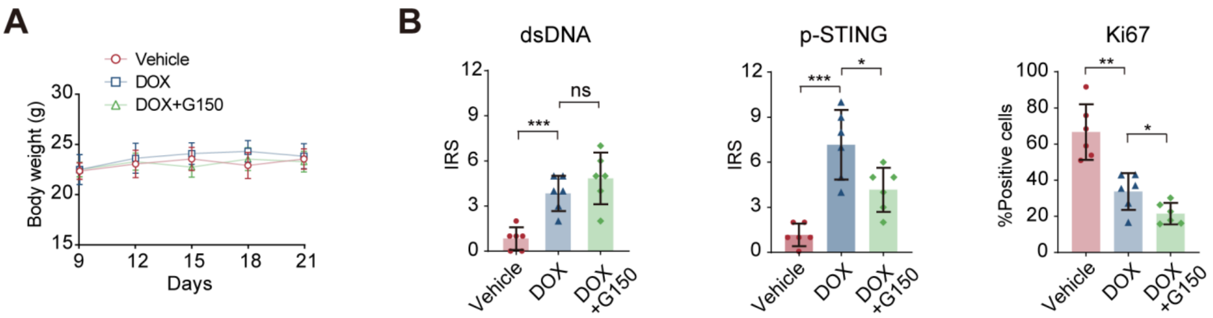

Figure S7. Chemotherapeutic agents could activate the cGAS-STING pathway, and cGAS inhibition enhances the tumor-killing effect in PTCL. A) Body weight change of mice in three groups during drug administration. (n = 6). B) Quantification analyses of dsDNA, p-STING, and Ki67 expression via IRS in three groups (Vehicle, DOX, and DOX plus G150 combination therapy) (n = 6). Student's t-test was used to detect differences between groups (Software GraphPad Prism 9.3). *****p<0.05, ******p<0.01, *******p<0.001, ns: not significant. DOX, doxorubicin; IRS: immunoreactivity score; dsDNA, double-stranded DNA; STING, stimulator of interferon genes; p-STING, phosphorylated STING
